# Supplementary material for: Protocol for a randomised controlled trial of Subacromial spacer for Tears Affecting Rotator cuff Tendons: a Randomised, Efficient, Adaptive Clinical Trial in Surgery (START:REACTS)
Source: BMJ Open. 2020 May 21;10(5):e036829. doi: 10.1136/bmjopen-2020-036829 (PMC7247380; doi:10.1136/bmjopen-2020-036829)
Supplement: Author's manuscript [file bmjopen-2020-036829.draft_revisions.pdf]

BMJ Open is committed to open peer review. As part of this commitment we make the peer review history of every article we publish publicly available.

When an article is published we post the peer reviewers' comments and the authors' responses online. We also post the versions of the paper that were used during peer review. These are the versions that the peer review comments apply to.

The versions of the paper that follow are the versions that were submitted during the peer review process. They are not the versions of record or the final published versions. They should not be cited or distributed as the published version of this manuscript.

BMJ Open is an open access journal and the full, final, typeset and author-corrected version of record of the manuscript is available on our site with no access controls, subscription charges or pay-per-view fees (http://bmjopen.bmj.com).

If you have any questions on BMJ Open's open peer review process please email info.bmjopen@bmj.com

BMJ Open

Protocol for a randomised controlled trial of Subacromial spacers for Tears Affecting Rotator cuff Tendons: a Randomised, Efficient, Adaptive Clinical Trial in Surgery (START:REACTS)

Journal:	*BMJ Open*
Manuscript ID	bmjopen-2020-036829
Article Type:	Protocol
Date Submitted by the Author:	06-Jan-2020
Complete List of Authors:	Metcalfe, Andrew; University of Warwick Warwick Medical School, Warwick Clinical Trials Unit; University Hospitals Coventry and Warwickshire NHS Trust, Trauma and Orthopaedics<br>Gemperle Mannion, Elke; University of Warwick Warwick Medical School, Warwick Clinical Trials Unit<br>Parsons, Helen; University of Warwick, Division of Health Sciences<br>Brown, Jaclyn; University of Warwick, Clinical Trials Unit; University of Warwick Clinical Sciences Research Institute, CTU<br>Parsons, Nicholas; University of Warwick, Warwick Medical School<br>Fox, Jo; Patient representative<br>Kearney, Rebecca; University of Warwick, Warwick Clinical Trials Unit<br>Lawrence, Tom; University Hospitals Coventry and Warwickshire NHS Trust, Trauma and Orthopaedics<br>Bush, Howard; University Hospitals Coventry and Warwickshire NHS Trust, Trauma and Orthopaedics<br>McGowan, Kerri; University Hospitals Coventry and Warwickshire NHS Trust<br>Khan, Iftekhar; University of Warwick Warwick Medical School, Centre for Health Economics at Warwick<br>Mason, James; University of Warwick, Warwick Medical School<br>Hutchinson, Charles; University of Warwick, Warwick Medical School<br>Gates, Simon; University of Birmingham, Cancer Research UK Clinical Trials Unit; University of Warwick, Clinical Trials Unit<br>Stallard, Nigel; University of Warwick,<br>Underwood, Martin; Warwick University, Warwick Medical School<br>Drew, Stephen; University Hospitals Coventry and Warwickshire NHS Trust, Trauma and Orthopaedics
Keywords:	Shoulder < ORTHOPAEDIC & TRAUMA SURGERY, Adult orthopaedics < ORTHOPAEDIC & TRAUMA SURGERY, Clinical trials < THERAPEUTICS

SCHOLARONE™
Manuscripts

I, the Submitting Author has the right to grant and does grant on behalf of all authors of the Work (as defined in the below author licence), an exclusive licence and/or a non-exclusive licence for contributions from authors who are: i) UK Crown employees; ii) where BMJ has agreed a CC-BY licence shall apply, and/or iii) in accordance with the terms applicable for US Federal Government officers or employees acting as part of their official duties; on a worldwide, perpetual, irrevocable, royalty-free basis to BMJ Publishing Group Ltd ("BMJ") its licensees and where the relevant Journal is co-owned by BMJ to the co-owners of the Journal, to publish the Work in this journal and any other BMJ products and to exploit all rights, as set out in our licence.

The Submitting Author accepts and understands that any supply made under these terms is made by BMJ to the Submitting Author unless you are acting as an employee on behalf of your employer or a postgraduate student of an affiliated institution which is paying any applicable article publishing charge ("APC") for Open Access articles. Where the Submitting Author wishes to make the Work available on an Open Access basis (and intends to pay the relevant APC), the terms of reuse of such Open Access shall be governed by a Creative Commons licence – details of these licences and which Creative Commons licence will apply to this Work are set out in our licence referred to above.

Other than as permitted in any relevant BMJ Author's Self Archiving Policies, I confirm this Work has not been accepted for publication elsewhere, is not being considered for publication elsewhere and does not duplicate material already published. I confirm all authors consent to publication of this Work and authorise the granting of this licence.

For peer review only

Protocol for a randomised controlled trial of Subacromial spacers for Tears Affecting Rotator cuff Tendons: a Randomised, Efficient, Adaptive Clinical Trial in Surgery (START:REACTS)

Authors:

Mr Andrew Metcalfe[1,2] <a.metcalfe@warwick.ac.uk> (corresponding author)

Mrs Elke Gemperle Mannion[2] <E.Gemperle-Mannion@warwick.ac.uk>

Dr Helen Parsons[2] <H.Parsons@warwick.ac.uk>

Ms Jaclyn Brown[2] <J.Brown.10@warwick.ac.uk>

Dr Nicholas Parsons[3] <nick.parsons@warwick.ac.uk>

Miss Josephine Fox[4] <josephine@bonesmart.org>

Dr Rebecca Kearney[1,2] <R.S.Kearney@warwick.ac.uk>

Mr Thomas Lawrence[1] <Tom.Lawrence@uhcw.nhs.uk>

Mr Howard Bush[1] <Howard.Bush@uhcw.nhs.uk>

Mrs Kerri McGowan[1] <kerri.mcgowan@uhcw.nhs.uk>

Dr Iftekhar Khan[3] <I.Khan.2@warwick.ac.uk>

Professor James Mason[2] <J.Mason@warwick.ac.uk>

Professor Charles Hutchinson[3] <C.E.Hutchinson@warwick.ac.uk>

Professor Simon Gates[5] <S.Gates@bham.ac.uk>

Professor Nigel Stallard[3] <N.Stallard@warwick.ac.uk>

Professor Martin Underwood[1,2] <M.Underwood@warwick.ac.uk>

Mr Stephen Drew[1] <Steven.Drew@uhcw.nhs.uk>

1. University Hospital of Coventry and Warwickshire NHS Trust, Coventry, UK
2. Warwick Clinical Trials Unit, University of Warwick, UK
3. Warwick Medical School, University of Warwick, UK
4. Patient member
5. University of Birmingham Clinical Trials Unit

For peer review only

For peer review only - http://bmjopen.bmj.com/site/about/guidelines.xhtml

Abstract

Introduction

Shoulder pain due to irreparable rotator cuff tears can cause substantial disability, but treatment options are limited. A balloon spacer is a relatively simple addition to a standard arthroscopic debridement procedure, but it is costly and there is no current randomised trial evidence to support its use. This trial will evaluate the clinical and cost effectiveness of the a subacromial balloon spacer for people undergoing arthroscopic debridement for irreparable rotator cuff tears.

New surgical procedures can provide substantial benefit to patients. Good quality randomised controlled trials (RCTs) are needed, but trials in surgery are typically long and expensive, exposing patients to risk and the healthcare system to substantial costs. One way to improve the efficiency of trials is with an adaptive sample size. Such methods are well established in drug trials but have rarely, if ever, been used in surgical trials.

Methods and analysis

START:REACTS is a participant and assessor blinded, adaptive, multi-centre RCT comparing arthroscopic debridement using the InSpace™ balloon (Stryker, USA) to arthroscopic debridement alone for people with symptomatic irreparable rotator cuff tears. It uses a group sequential adaptive design where interim analyses are performed utilising all of the three, six and twelve month data that are available at each time point. A maximum of 221 participants (1:1 ratio) will be required to provide 90% power for a 10 point difference in the primary outcome; the Constant Score at 12 months. A sub-study will use deltoid-active MRI scans in 56 participants to assess the function of the balloon. Analysis will be on an intention-to-treat basis and reported according to principles established in the CONSORT statement.

Ethics and dissemination

NRES number 18/WM/0025. The results will be disseminated via peer-reviewed publications, presentations at conferences, lay summaries and social media.

Trial Registration number: ISRCTN17825590.

For peer review only - http://bmjopen.bmj.com/site/about/guidelines.xhtml

Keywords

Randomised Controlled Trial; Shoulder; Rotator Cuff; Subacromial spacer balloon; adaptive design;

Article Summary

Strengths and limitations of this study

- Multi-centre randomised trial of a subacromial spacer balloon following debridement, compared to debridement alone, for irreparable rotator cuff tears of the shoulder.
- Participant-assessor blinding, including blinded operation notes with novel unblinding mechanism.
- Mechanistic MRI sub-study of 56 participants with images at eight weeks and six months after surgery.
- Statistical adaptive design, with hard stopping rules for futility or efficacy based on emerging outcomes.

For peer review only - http://bmjopen.bmj.com/site/about/guidelines.xhtml

INTRODUCTION

Subacromial spacer balloons

Shoulder pain is a common and disabling problem. The UK population prevalence of shoulder pain is approximately 16%.(1) Rotator cuff disease accounts for 70% - 85% of this.(2-4) People with a symptomatic rotator cuff tear typically have pain, restricted movement, loss of strength and disability. The condition is associated with substantial expense to society through both costs of treatment and loss of work (both paid and unpaid).(5-8) Rotator cuff repair is a widely accepted treatment for symptomatic rotator cuff tears.(9, 10) Some tears cannot be surgically repaired, these are called irreparable tears.

Symptomatic irreparable rotator cuff tears are a challenging condition to treat. Treatment options including physiotherapy, injections, arthroscopic debridement, partial repair, muscle transfers, interposition grafts and even shoulder replacements.(11-14) Arthroscopic debridement is commonly used and case-series suggest it may be beneficial, but it remains a controversial option, with little randomised controlled trial data on its use in the irreparable tear population.(15-17)

In 2013**,** the InSpace subacromial balloon spacer (Stryker, Michigan, USA) was introduced into UK orthopaedic practice as a potential treatment option for people with irreparable tears of the rotator cuff. In May 2016 an interventional procedure guidance document was published by the UK National Institute for Health and Care Excellence (NICE), which found that there was very limited evidence for its use. Therefore, the device was limited to research use only and a recommendation was made to assess its effectiveness.(18)

The InSpace device is a saline-filled balloon made of biodegradable (dissolvable) synthetic material. It is inserted above the main joint of the shoulder at the end of an arthroscopic debridement after an irreparable tear has been identified. It is simple to deploy and adds relatively little time to the operation.(19, 20) It cushions the humerus from pressing on the bone above it (the acromion) when the deltoid is active and during abduction of the arm, potentially reducing pain. It may also assist in the biomechanics of the shoulder, resisting proximal migration of the humerus under deltoid activity. The device begins to degrade and deflate from three months after surgery. During this time, it is thought to improve rehabilitation of the remaining rotator cuff and deltoid, so that when the device deflates, the biomechanics of the shoulder are better preserved.

The safety of the device in rodents has been established, with only one adverse event: a fibrosarcoma that was thought to be unique to rodents.(21) Proof of concept has been established in a series of 24 cases performed for irreparable cuff tears in Slovakia in 2012, with 5-year follow-up results published in 2016.(22, 23) The device has been used in a number of centres across the UK, with multiple conference abstracts from the UK.(24-26) These have demonstrated improvements in outcomes from baseline. Complications such as balloon displacement and non-cyst forming synovitis have been reported in a small number of cases (3 out of 61). One retrospective non-randomised study of 23 patients (12 with the balloon) showed an improvement in outcomes compared to debridement alone (27). There are no completed randomized controlled trials (RCTs).

For peer review only - http://bmjopen.bmj.com/site/about/guidelines.xhtml

The benefits found in case series may not be unique to the InSpace balloon, although the relative effectiveness of the balloon in comparison to non-operative care or acromioplasty is not known and may give substantial improvements. (17)

There is one other RCT in progress for this device – a company funded study in the USA which will recruit 184 participants, comparing partial cuff repair with balloon as a stand-alone intervention (clinicaltrials.gov NCT02493660). Results are expected in mid-2020. Partial cuff repair is not a technique that is widely used in the UK and is not an appropriate comparator in a UK context.

Trial designs in new surgical procedures

The safe introduction of new surgical procedures is essential to the delivery of high quality surgical care for patients. New procedures can result in a step-change improvement in treatment, but also introduce new risks, and substantial costs. Major harm can be done when a misunderstood or well-intentioned intervention is used widely across the health service before it is formally evaluated. (28, 29)

While pharmaceuticals must undergo rigorous clinical trials before introduction, this is not the case for surgical procedures.(30) When surgical procedures are assessed rigorously, studies tend to be large multi-centre, randomised trials which typically need to recruit over extended time periods.(29, 31) Improvements in the efficiency of delivering trials of surgical interventions would provide earlier answers to these important clinical questions, providing benefits to patients and making better use of healthcare resources.

The use of adaptive trial designs has been encouraged by major journals, the US Food and Drug Administration, and National Institute of Health Research (NIHR) panels.(32-34) Adaptive trial designs allow prospectively planned modifications, such as stopping the study early or dropping an intervention, based on emerging findings as the trial proceeds, while preserving the scientific validity and integrity of the trial. This flexible strategy typically reduces costs and shortens time-scales, without compromising the integrity, statistical power or rigour of the study.(32, 35-37)

In this trial we are using a novel statistical adaptive design approach for the assessment of a new surgical procedure. The statistical principles are laid out in a 2019 methodology paper.(38) Briefly, the design uses early (three and six month Constant score) endpoint data to support emerging outcome data for the primary endpoint (12 month Constant score) to make decisions about stopping for either futility or efficacy at pre-planned interim analyses that occur before the end of recruitment. In the current study, we will apply these design principles to a multi-centre clinical trial of a new surgical procedure.

Aim

To assess the clinical-effectiveness, cost-effectiveness and safety of a sub-acromial spacer balloon for patients with symptomatic irreparable tears of the rotator cuff.(18)

For peer review only - http://bmjopen.bmj.com/site/about/guidelines.xhtml

METHODS

Trial Design

The Subacromial spacer for Tears Affecting Rotator cuff Tendons (START) study is a participant and assessor blinded, adaptive, multi-centre RCT based in the UK comparing arthroscopic debridement using the InSpace balloon to arthroscopic debridement alone, performed using the REACTS (Randomised, Efficient, Adaptive Clinical Trial in Surgery) framework. Patient involvement has been a core to the design and delivery of the study, and will remain so, including in the interpretation and dissemination of results.

Objectives

Clinical objectives

- Our **primary clinical objective** is to quantify and draw inferences on observed differences between arthroscopic debridement of the subacromial space and arthroscopic debridement with insertion of the InSpace balloon (Stryker, Michigan, USA) 12 months after surgery, using the Constant score as the primary outcome measure (39, 40).

Secondary clinical objectives:

- To quantify and draw inferences on observed differences between arthroscopic debridement and arthroscopic debridement with insertion of the InSpace balloon (Stryker, Michigan, USA) on: Shoulder function; patient reported outcome measures; adverse events and; resource use at three, six and 12 months.

- To perform an economic analysis, assessing the comparative cost-effectiveness of the two treatments.

- To compare the acromio-humeral distance on MRI scans in a sample of participants with and without the balloon at eight weeks and six months after treatment, to assess the proposed mechanism of action of the balloon when it is still inflated (at eight weeks) and to determine if the effect persists when it has deflated (at six months).

Methodological Objectives

- The **primary methodological objective** is to develop and implement appropriate statistical tools to allow an efficient adaptive clinical trial design, with the potential for early stopping for either futility or efficacy, using Constant score data available at three, six and 12 months.(38)

Ethics, registration and oversight

The trial will be conducted in full conformance with the principles of the Declaration of Helsinki and to MRC Good Clinical Practice (GCP) guidelines. It will also comply with all applicable UK legislation and University of Warwick Standard Operating Procedures (SOPs). Trial oversight is provided by a Data Monitoring Committee (DMC) and Trial Steering

For peer review only - http://bmjopen.bmj.com/site/about/guidelines.xhtml

Committee (TSC), both are made up of a majority of independent members and are conducted according to Warwick SOPs. Monitoring and audit will be undertaken by the sponsors according to a monitoring plan. The trial received full research ethical approval (RES number 18/WM/0025) on 13th February 2018 and was registered on ISRCTN on the 6th April 2018 (ISRCTN17825590), prior to commencing recruitment which is ongoing. Amendments will be communicated to sites by the co-ordinating team.

Outcome measures

Primary outcome: The Constant score at 12 months.(39-41) It is a widely used outcome score in shoulder trials, is well accepted by surgeons and has good reliability and responsiveness when used with standardised protocols.(17, 22, 39-45) Based on our meta-analysis of outcomes for randomised trials, the Constant score typically reaches a plateau by 12 months after any intervention for a rotator cuff tear.(46) The 24 month Constant score does not give sufficient additional value to justify the increase in costs that would be required for its collection.

A standardised protocol for the objective component of the score has been developed based on the work of Moeller *et al.* with training provided for all sites.(41) Participants will attend in person for measurements either for research visits or alongside their routine clinical assessment. The strength measurements will be collected using a supplied IsoForceControl EVO2 dynamometer (Herkules Kunstoff, Switzerland) and the range of motion will be collected using a long-handled goniometer (12 ½").

Secondary outcomes:

- The Constant Score at baseline, three, and six months. (39, 40)
- Range of pain-free movement of the shoulder at baseline, three, six and 12 months measured using a long-arm goniometer.
- Strength of shoulder abduction and flexion at baseline, three, six, and 12 months measured using the same supplied dynamometer.
- The Oxford Shoulder Score (OSS) at baseline, three, six, 12 and 24 months.(47).
- Western Ontario Rotator Cuff index at baseline, three, six, 12 and 24 months.(48, 49)
- Health utility assessed using EQ-5D-5L at baseline, three, six and 12 months.(50, 51)
- Healthcare resource collected at three, six, and 12 months
- Patient global assessment of change (PGIC) score, collected at three, six, and 12 months. A simple seven-point scale assessing perception of improvement.(52)
- Analgesia use (drug and approximate frequency) collected at baseline and three, six and 12 months.

For peer review only - http://bmjopen.bmj.com/site/about/guidelines.xhtml

- MRI Scans (sub-study of 56 patients, six weeks and six months post-surgery): see 'MRI sub-study' below.

- Adverse events will be collected from site reports and from participants in the 3, 6 and 12 months questionnaires.

Patient reported outcome measures (OSS, WORC, EQ-5D-5L, PGIC) will be collected at 24 months as a secondary outcome, these will be published separately and will not delay publication of the primary 12 month outcome data). A detailed data management plan has been prepared following Warwick SOPs and will be available on request.

Eligibility criteria

Inclusion criteria

1. Rotator cuff tear deemed by the treating clinician to be technically irreparable*. Many factors other than size influence whether a tear can be repaired (such as chronicity, retraction of the tendon ends, fat infiltration in muscle). However, a potential participant who has a tear that is technically repairable, such as a small tear, but is unsuitable for repair due to age or co-morbidities, is not eligible for this study.

2. Intrusive symptoms (pain and loss of function) which in the opinion of the treating clinician warrants surgery.

3. Non-operative management has been unsuccessful. The exact nature of non-operative management will be left for the treating clinician to decide.

Exclusion criteria

4. Advanced gleno-humeral osteoarthritis (OA) on pre-operative imaging (in the opinion of the treating clinician). Advanced gleno-humeral OA may be interpreted as Kellgren Lawrence grade 3 or 4 changes on routine pre-operative radiographs(53), or the MRI equivalent if radiographs have not been taken.

5. Subscapularis deficiency*, defined as a tear involving more than the superior 1cm (approximately) of the subscapularis if repaired, or any tear that is not repaired.

6. The treating clinician determines that interposition grafting or tendon transfers are indicated.

7. Pseudoparalysis (an inability to actively abduct or forward flex up to 20°), as determined by the treating clinician.

8. Unrelated, symptomatic ipsilateral shoulder disorder that would interfere with strength measurement or ability to perform rehabilitation

For peer review only - http://bmjopen.bmj.com/site/about/guidelines.xhtml

9. Other neurological or muscular condition that would interfere with strength measurement or ability to perform rehabilitation, in the opinion of the treating clinician.

10. Previous proximal humerus fracture that could influence shoulder function, as determined by the treating clinician.

11. Previous entry into the present trial (i.e. other shoulder).

12. Unable to complete trial procedures.

13. Age under 18

14. Unable to consent to the trial.

15. Unfit for surgery as defined by the treating clinician.

*criteria regarding whether the tear is technically repairable, and the integrity of the subscapularis are unreliably assessed by pre-operative imaging and will be reassessed in theatre, prior to randomisation. People not eligible to be enrolled in the trial will be treated according to the best judgement of the surgeon at the time.

Participant identification / Screening

Potential participants will be identified by the attending clinical team by clinicians in intermediate or secondary care clinics, or from the surgical waiting list. The attending clinician will confirm appropriateness for study eligibility based on clinical assessment and standard care pre-operative imaging for that site.

All potential participants who meet the study entry criteria will be checked for eligibility and recorded on the monthly screening log. Potential participants who are willing to be approached by a suitably trained member of the research team will be provided with verbal and written information about the study, and will have the opportunity to discuss and ask questions about the study prior to informed consent being obtained.

Eligibility for the study is confirmed by the operating surgeon intra-operatively and patients may be excluded at this stage if the surgeon finds that the rotator cuff tear can be repaired. These excluded participants will be informed by letter that they are no longer taking part in the study. Baseline data will be retained to explore any differences between those who were deemed ineligible at surgery to those who were randomised.

Randomisation

Participants are randomly allocated on a 1:1 basis to the two treatment groups via a central computer-based randomisation system provided by Warwick Clinical Trials Unit (WCTU, independent of the study team). A minimisation algorithm is used to determine participant allocation, using site, gender, age group (<70 years and ≥70 years) and cuff tear size (as assessed by the operating surgeon, ≥3cm or <3cm) as factors, with a random element

For peer review only - http://bmjopen.bmj.com/site/about/guidelines.xhtml

included to provide a 70% chance that the participant will receive the treatment that minimises the imbalance, to ensure unpredictability.

Randomisation will be performed by theatre staff, after the intra-operative findings have been checked (including cuff tear size) and eligibility is confirmed. Staff use an online system in a separate room to maintain blinding, with a 24 hour back-up automated telephone system available.

Trial treatment(s) / intervention

Standard Arthroscopic debridement (control): The control group will be an arthroscopic debridement of the subacromial space with removal of inflamed tissue (bursectomy) and unstable remnants of the torn tendon, limited bone resection of the acromion, retention of the coraco-acromial (CA) ligament, and biceps tenotomy (if not already torn). The anaesthetic will be left to the choice of the anaesthetist; this may include general or local anaesthesia. Surgeons may use their normal surgical technique, within the confines described in a trial specific surgical guideline (available at the trial website or at request from Warwick CTU).

Group 2 – Standard Arthroscopic debridement plus insertion of InSpace balloon (Intervention): Arthroscopic debridement, as described above, with insertion of the InSpace Balloon performed by sub-speciality trained shoulder surgeons. The same arthroscopic debridement will be performed as described for the control group. If allocated to the balloon procedure, the company's recommended surgical technique will be followed for sizing, insertion and deployment of the balloon, as documented in the surgical manual.

For both groups, fidelity will be assessed with an operative record form and arthroscopic photographs taken at the end of debridement and just before balloon inflation.

Rehabilitation

Post-operative rehabilitation for both groups will be blind to treatment allocation and will include standardised post-operative information, home exercises and the offer of a minimum of three face-to-face physiotherapy appointments. Additional physiotherapy will be at the discretion of the trial sites. A physiotherapy trial manual will be followed to standardise rehabilitation progression. All materials were developed through a process of collecting current NHS protocols, manufacturer protocols, scoping the literature and expert consensus.

Blinding

Treatment allocation will be blinded for both patients and assessors, only the surgical teams at the time of the operation will be aware of the allocation. Theatre staff are asked not to discuss the balloon and to communicate the allocation by using methods such as holding up a piece of paper on which the allocation is clearly written. If participants are awake, drapes are used to obscure the participant's view and arthroscopic screens will be positioned in such a way that the patient is unable to see the procedure.

For peer review only - http://bmjopen.bmj.com/site/about/guidelines.xhtml

The incisions required for the two operations are similar and there is no external way in which the patients will be able to detect the presence or absence of the balloon, except for the size of the lateral portal. One of the incisions (the lateral portal) will need to be slightly larger to insert the balloon – 1.5cm as opposed to 1cm. A 1.5cm incision will be used for all participants, which is a very small change from standard care and is very unlikely to have a negative effect on any participant. Incisions will therefore be the same for both groups.

The operation note will be blinded to prevent accidental unblinding of the patient (for example, in the discharge information, or during post-operative physiotherapy). A standard recommended operation note template will be given to all sites adjusted to fit their local operation note systems. The details of the operation related to the balloon will be recorded in a secure online form easily accessible to the surgeon.

Unblinding may very rarely be required in an emergency situation, such as an overnight admission for suspected post-operative infection. Unblinding will be performed only by NHS staff in an emergency situation, by using a pre-defined web-based system, from a link inserted in the operation note. A two-way secure verification process will be performed using email, and an access code will be emailed only to an active NHS email address. A full explanation of the clinical circumstances and the need for access to data will be requested by the trial team for audit and monitoring purposes from the person who performed the unblinding, and the principal investigator (PI) for the site will be informed. The system has been designed and tested by the WCTU programming team to ensure that it is both secure and fully functional.

Participants will be asked at the 12 month time point, after collection of the primary outcome, if they were aware of their allocation.

End of trial

The trial will end when all participants have completed their 24 month follow-up, but the results will be published after the 12 month outcomes have been analysed, with a secondary report after collection of 24 month outcomes.

The trial may be stopped prematurely if: 1) mandated by the Ethics Committee; 2) There is an unexpected major safety concern; 3) Following recommendations from the Data Monitoring Committee (DMC) or Trial Steering Committee (TSC), which may include meeting the adaptive design boundaries, or 4) Funding for the trial ceases

Once a decision to stop has been taken, those within a short waiting time to surgery may be randomised at the discretion of the DMC and TSC, but after that participants will be treated according to the judgement of their clinician.

MRI Sub-study

Participants selected for inclusion in the MRI sub-study will undergo two research MRI scans at eight weeks and six months post intervention. This will assess the mechanism of action of the balloon, when the balloons are likely to be still inflated (but when acute post-operative pain has subsided), and when they are likely to have fully deflated, to see if the proposed

For peer review only - http://bmjopen.bmj.com/site/about/guidelines.xhtml

mechanism for ongoing improvement is maintained. Passive imaging alone will not be adequate to demonstrate the function of the balloon and imaging will also need to be performed when the deltoid muscle is active, producing a proximally directed force on the humerus.(54, 55)

For this study we have developed (and piloted) a novel dynamic approach to assessing the function of the rotator cuff using MRI imaging under a mild deltoid load, to specifically assess the mechanism of the balloon. A separate paper on the pilot study for the trial is in preparation and will describe these methods in more detail.

The primary outcome will be the minimum acromio-humeral distance (AHD) on the 'deltoid-active' coronal sequences at six months, a reliable and proven measure. (56-58) Secondary measures will be AHD on passive images, and the change in AHD between active and passive images. The position of the balloon will be assessed to check for migration and consistency of placement relative to the acromion.

Adverse Events (AE), Adverse Device Events (ADE), Serious Adverse Device Effects (SADE)

Adverse events (AE), serious adverse events (SAEs), adverse device effects (ADE) and serious adverse device effects (SADE) will be defined using standard accepted criteria. An Unanticipated Serious Adverse Device Effect (USADE) is defined as a SADE which by its nature, incidence, severity or outcome has not been identified in the current version of the risk analysis report.

For the purposes of this trial AEs will be recorded for any participant where it is thought there may be a relationship between the trial interventions or the condition being studied (in this case, any shoulder condition, or related to the anaesthetic).

All SAEs SADEs and USADEs occurring from the time of randomisation until 12 months post-randomisation must be communicated to the sponsor within 24 hours of the research staff becoming aware of the event. Events will be followed up until the event has resolved or a final outcome has been reached.

Where participants have been lost to follow up at or beyond the 12 month time point, and data on adverse events can therefore not be recorded, the participants General Practitioner (GP) will be contacted and a short form requesting any information or health record that could be an adverse event will be requested from the GP, as well as confirmation of the current contact details of the participant.

STATISTICAL ANALYSIS

Power and sample size

The target difference that has been chosen for the Constant Score is 10 units, as has been widely used for other trials.(17, 59, 60) Typically in other studies of this population the standard deviation has been taken to be 20, giving a moderate standardised mean difference of 0.5.(17, 22) For a costly invasive procedure of this nature a smaller

For peer review only - http://bmjopen.bmj.com/site/about/guidelines.xhtml

standardised mean difference is unlikely to be considered worthwhile. For a power of 90% and (two-sided) type I error rate of 5% a study with a conventional fixed design (i.e. with no possibility of stopping early) would require 170 participants.

In order to assess the design characteristics and estimate the required sample size for the planned adaptive design, we undertook a large simulation study (see Parsons et al.(38)). The simulations showed that an adaptive design that allowed the possibility of early stopping for efficacy and/or futility, was feasible for the START:REACTS study.

Full details of the stopping boundaries, and the timing of the interim analyses, will not be provided here in order to ensure trial integrity, these will remain confidential and will be shared only between the study statistician and the DMC. Based on an assumed modest correlation between three and six month Constant scores and 12 month Constant score equal to 0.5, and standard deviations of 20 for both three and six month scores, the simulations for the selected adaptive design, indicated that 188 participants would be sufficient to detect a 10 point difference in Constant score between treatment arms with 90% power, and 5% (two-sided) type I error rate. Allowing for 15% loss to follow-up, whilst striving to keep this below 10%, gives a maximum study sample size of 221.

The sample size for MRI sub-study is based on the study by Gumina *et al.*(56), where the minimum acromio-humeral distance had a standard deviation of 1.72mm. To observe a minimum important difference of 1.5mm (larger than the minimum detectable change of 1.3mm established elsewhere(61)) with an alpha of 0.05 at 80% power, assuming a loss to follow-up at 6 months of 20%, requires 56 participants for the MRI sub-study.

Statistical analysis plan

All data will be analysed and reported in accordance with the Consolidated Standards of Reporting Trials (CONSORT) guidelines.(62, 63). A detailed statistical analysis plan and a data sharing plan will be agreed with the DMC prior to any formal analyses being conducted.

Baseline data will be summarised to check comparability between treatment arms. Standard statistical summaries (e.g. means and standard deviations, dependent on data type) will be presented for the primary outcome measure and all secondary outcome measures.

The primary analysis will investigate differences in the Constant score 12 months after surgery between the two treatment groups. It will be conducted on an intention-to-treat basis following the methods, test statistics and boundaries described by Parsons *et al*.(38) To preserve the integrity of the study, the exact boundaries used for testing are given in the adaptive charter known only to the study team and independent DMC. Briefly, if the study recruits to target, the method set out by Parsons *et al*.(38) will be used to calculate the boundaries and if the study stops early, testing will proceed using boundaries calculated by the deletion method for overrunning analysis, described by Whitehead, with clinical inferences following directly from widely used methods for unbiased estimates and confidence intervals in group sequential trials. (64-66)

The primary analysis will be augmented by calculating adjusted estimates of treatment group differences (with 95% confidence intervals) for the Constant score using a mixed-effects model. The mixed-effects model will include a random effect for the recruiting

For peer review only - http://bmjopen.bmj.com/site/about/guidelines.xhtml

centre, and fixed effects for the variables of interest included in the minimisation algorithm, patient age, gender and size of tear. In addition, subject to the observed data, sensitivity analyses such as per-protocol analyses or multiple imputation will also be undertaken. If undertaken, all multiple imputation assumptions will be justified and reported. Estimates of efficacy for the other outcome measures (for example Oxford Shoulder Score) will follow this approach to analysis.

Pre-specified sub-group analyses will be undertaken to assess whether there is evidence that the intervention effect differs with respect to:

- The size of the rotator cuff tear as measured at the start of surgery, defined as large or massive cuff tear (≥3cm) or moderate to small (<3cm).
- Gender
- Age (>70 or <70)

These have been chosen as they are either important to the function of the intervention (cuff tear size) or to interpretation of the Constant score (gender and age). The subgroup analyses will follow the methods described for the mixed effects model of the Constant score above, with additional interaction terms incorporated into the mixed-effects regression model to assess the level of support for these hypotheses. The study is not powered to formally test these hypotheses, so they will be reported as exploratory analyses only, and as subsidiary to the analysis reporting the main effects of the intervention in the full study population(67).

Interim analyses will only be undertaken following the principles laid out by Parsons et al.(38) Details of the timings or settings for the interim analyses will be treated as confidential information, so as not to prejudice the outcome of the trial based on the decision to stop the study or proceed, but will be recorded in the DMC minutes and on date-stamped internal documents. If a decision to stop the study early is made (for reasons other than safety), it will not be communicated outside of the DMC closed meeting whether this is for efficacy or futility.

HEALTH ECONOMIC EVALUATION

The economic component will include a standard health policy-relevant economic analysis and an exploration of how early economic data can be used to support decision making through the use of an adaptive design.

A prospective economic evaluation will be integrated into the trial and adhere to the recommendations of the NICE Reference Case.(68) Mechanisms of missingness of data will be explored and multiple imputation methods will be applied to impute missing data. Imputation sets will be used in bivariate analysis of costs and QALYs to generate incremental cost per QALY estimates and confidence regions.(69-72) It is anticipated that incremental costs and benefits will be captured within the trial and that extrapolated modelling will not be required.

Relatively little research has been conducted on how economic data available at interim time points can inform interim decision making. Currently, methods used are based mainly on net monetary benefit (NMB) approaches using value of information (VOI) methods (73,

For peer review only - http://bmjopen.bmj.com/site/about/guidelines.xhtml

74). We will compare various methods using economic data (costs and QALYs) and clinical data to evaluate the practical implications and operating characteristics of stopping a trial early based on cost-effectiveness data alone, efficacy data alone or a combination of cost-effectiveness and efficacy. We will use the trial to evaluate putative analytic methods, as set out within a prospectively written health economic analysis plan and carry out parallel interim analyses, separate from the real trial analyses, exploring how interim decisions might have been influenced. As such health economic decision rules for adaptive designs are less widely understood and used in the literature, the properties of these methods will be developed further for future trials.

DISSEMINATION AND PUBLICATION

The trial will be reported in accordance with the Consolidated Standards of Reporting Trials (CONSORT) guidelines and the 2018 CONSORT extension for adaptive trials.(62, 63)

The results will be submitted to a high impact peer-reviewed journal with authorship following ICMJE recommendations, and will be presented at national and international meetings such as the British Elbow and Shoulder Society, the British Orthopaedic Association, and the American Academy of Orthopaedic Surgeons. The investigators will share data (with associated coding library) used in developing the results on request to the chief investigator, subject to a formal data sharing agreement being in place. We will only share anonymised record level data with those who have received ethical clearance from their host institution.

To inform patients and the public, we intend to produce a lay summary, which will be made available in the trial hospitals and to patients involved in the trial. In addition, we will publicise the work through social media outlets (e.g. Facebook and twitter) as well as websites such as Patient.co.uk.

CONCLUSION

In this protocol paper, a trial is presented of a NICE research priority, to assess the clinical and cost-effectiveness of a subacromial spacer balloon device for the treatment of irreparable tears of the rotator cuff. A novel adaptive group sequential design will be used, with early stopping supported by all of the available three, six and 12 month Constant score data at two interim analysis time-points. A MRI sub-study will be performed on 56 participants to assess the proposed mechanism of the balloon. Robust methods have been developed to ensure the study is conducted to the highest standards, including blinding of operation notes and a novel method for unblinding in case of emergency. The study is expected to report in February 2022, or earlier depending on whether the study runs to its maximum length.

For peer review only - http://bmjopen.bmj.com/site/about/guidelines.xhtml

For peer review only

For peer review only - http://bmjopen.bmj.com/site/about/guidelines.xhtml

Author Contributions. All the authors helped to developed the trial protocol and contributed to the writing of the manuscript. This protocol was written following the SPIRIT protocol guidance. Andrew Metcalfe is the chief investigator and main grant holder for this study.

Funding. This project (project reference 16.61.18) is funded by the Efficacy and Mechanism Evaluation (EME) Programme, an MRC and NIHR partnership. The views expressed in this publication are those of the author(s) and not necessarily those of the MRC, NIHR or the Department of Health and Social Care.

Sponsorship. The trial is co-sponsored by the University of Warwick and University Hospitals Coventry and Warwickshire NHS trust.

Competing interests. Stryker are providing 50 free InSpace balloons for the study, to offset some of the costs of delivery of the study in participating hospitals. Contracts are in place to ensure the full independence of the trial team with regard to study design and delivery, analysis and reporting of results, aligning to the NIHR standard agreement. The chief investigator and multiple co-authors (HP, EGM, JB, JF, JM, CH. MU) are applicants on another NIHR grant with similar contractual relationships with Stryker to support treatment costs but not research costs, in which the full independence of the trial team is protected by mutually agreed contracts.

MU was Chair of the NICE accreditation advisory committee until March 2017 for which he received a fee. He is chief investigator or co-investigator on multiple previous and current research grants from the National Institute for Health Research. He is an NIHR Senior Investigator. He is an editor of the NIHR journal series, and a member of the NIHR Journal Editors Group, for which he receives a fee.

There are no other conflicts of interest to declare

Provenance and peer review. Not commissioned; peer reviewed for ethical and funding approval prior to submission.

Open Access. This is an Open Access article distributed in accordance with the terms of the Creative Commons Attribution (CC BY 4.0) license, which permits others to distribute, remix, adapt and build upon this work, for commercial use, provided the original work is properly cited. See: http://creativecommons.org/licenses/by/4.0/

For peer review only

For peer review only - http://bmjopen.bmj.com/site/about/guidelines.xhtml

REFERENCES

1. Carnes D, Parsons S, Ashby D, Breen A, Foster NE, Pincus T, et al. Chronic musculoskeletal pain rarely presents in a single body site: Results from a UK population study. Rheumatology. 2007;46:1168-70.
2. Ostor AJ, Richards CA, Prevost AT, Speed CA, Hazleman BL. Diagnosis and relation to general health of shoulder disorders presenting to primary care. Rheumatology (Oxford). 2005;44(6):800-5.
3. Teunis T, Lubberts B, Reilly BT, Ring D. A systematic review and pooled analysis of the prevalence of rotator cuff disease with increasing age. J Shoulder Elbow Surg. 2014;23(12):1913-21.
4. Urwin M, Symmons D, Allison T, Brammah T, Busby H, Roxby M, et al. Estimating the burden of musculoskeletal disorders in the community: the comparative prevalence of symptoms at different anatomical sites, and the relation to social deprivation. Ann Rheum Dis. 1998;57(11):649-55.
5. Largacha M, Parsons IMt, Campbell B, Titelman RM, Smith KL, Matsen F, 3rd. Deficits in shoulder function and general health associated with sixteen common shoulder diagnoses: a study of 2674 patients. J Shoulder Elbow Surg. 2006;15(1):30-9.
6. Minns Lowe CJ, Moser J, Barker K. Living with a symptomatic rotator cuff tear 'bad days, bad nights': a qualitative study. BMC Musculoskelet Disord. 2014;15:228.
7. Virta L, Joranger P, Brox JI, Eriksson R. Costs of shoulder pain and resource use in primary health care: a cost-of-illness study in Sweden. BMC Musculoskelet Disord. 2012;13:17.
8. Whittle S, Buchbinder R. In the clinic. Rotator cuff disease. Ann Intern Med. 2015;162(1):ITC1-15.
9. Carr AJ, Cooper CD, Campbell MK, Rees JL, Moser J, Beard DJ, et al. Clinical effectiveness and cost-effectiveness of open and arthroscopic rotator cuff repair [the UK Rotator Cuff Surgery (UKUFF) randomised trial]. Health Technol Assess. 2015;19(80):1-218.
10. Acevedo DC, Paxton ES, Williams GR, Abboud JA. A Survey of Expert Opinion Regarding Rotator Cuff Repair. J Bone Joint Surg Am. 2014;96(14):e123.
11. Anley CM, Chan SK, Snow M. Arthroscopic treatment options for irreparable rotator cuff tears of the shoulder. World J Orthop. 2014;5(5):557-65.
12. Leung B, Horodyski M, Struk AM, Wright TW. Functional outcome of hemiarthroplasty compared with reverse total shoulder arthroplasty in the treatment of rotator cuff tear arthropathy. J Shoulder Elbow Surg. 2012;21(3):319-23.
13. Harreld KL, Puskas BL, Frankle M. Massive rotator cuff tears without arthropathy: when to consider reverse shoulder arthroplasty. J Bone Joint Surg Am. 2011;93(10):973-84.
14. Mulieri P, Dunning P, Klein S, Pupello D, Frankle M. Reverse shoulder arthroplasty for the treatment of irreparable rotator cuff tear without glenohumeral arthritis. J Bone Joint Surg Am. 2010;92(15):2544-56.
15. Rockwood CA, Jr., Williams GR, Jr., Burkhead WZ, Jr. Debridement of degenerative, irreparable lesions of the rotator cuff. J Bone Joint Surg Am. 1995;77(6):857-66.
16. Liem D, Lengers N, Dedy N, Poetzl W, Steinbeck J, Marquardt B. Arthroscopic debridement of massive irreparable rotator cuff tears. Arthroscopy. 2008;24(7):743-8.
17. Kukkonen J, Joukainen A, Lehtinen J, Mattila KT, Tuominen EK, Kauko T, et al. Treatment of non-traumatic rotator cuff tears: A randomised controlled trial with one-year clinical results. Bone Joint J. 2014;96-B(1):75-81.

For peer review only - http://bmjopen.bmj.com/site/about/guidelines.xhtml

18. National Institute for Health and Care Excellence. Biodegradable subacromial spacer insertion for rotator cuff tears. Interv Proced Guid IPG558 [Internet]. 2016 11/04/17. Available from: https://www.nice.org.uk/guidance/ipg558.
19. Savarese E, Romeo R. New solution for massive, irreparable rotator cuff tears: the subacromial "biodegradable spacer". Arthrosc Tech. 2012;1(1):e69-74.
20. Szollosy G, Rosso C, Fogerty S, Petkin K, Lafosse L. Subacromial spacer placement for protection of rotator cuff repair. Arthrosc Tech. 2014;3(5):e605-9.
21. Ramot Y, Nyska A, Markovitz E, Dekel A, Klaiman G, Zada MH, et al. Long-term Local and Systemic Safety of Poly(L-lactide-co-epsilon-caprolactone) after Subcutaneous and Intra-articular Implantation in Rats. Toxicol Pathol. 2015;43(8):1127-40.
22. Senekovic V, Poberaj B, Kovacic L, Mikek M, Adar E, Dekel A. Prospective clinical study of a novel biodegradable sub-acromial spacer in treatment of massive irreparable rotator cuff tears. Eur J Orthop Surg Traumatol. 2013;23(3):311-6.
23. Senekovic V, Poberaj B, Kovacic L, Mikek M, Adar E, Markovitz E, et al. The biodegradable spacer as a novel treatment modality for massive rotator cuff tears: a prospective study with 5-year follow-up. Archives of Orthopaedic and Trauma Surgery. 2016;137:95-103.
24. Bakti N, Wlliamson M, Gulihar M, Singh R. Patient reported functional outcome following subacromial spacer insertion for irrepairable rotator cuff tears: a 12 month review. BJJ Orthop Proc. In press.
25. Henderson D, Simeson K, Venkateswaran B. Sub-acromial spacer vs. partial rotator cuff repair; A single surgeon cohort study. BJJ Orthop Proc. In press.
26. Prasad VR, Fung M, Borowsky KA, Tolat AR, Singh B. Outcomes of inspace balloon arthroplasty for irreparable cuff tear at two years: a longitudinal study. Bone & Joint Journal Orthopaedic Proceedings Supplement. 2016;98-B(SUPP 13):1-.
27. Holschen M, Brand F, Agneskirchner JD. Subacromial spacer implantation for massive rotator cuff tears. Obere Extremität. 2017;12:38-45.
28. Costa ML, Achten J, Parsons NR, Edlin RP, Foguet P, Prakash U, et al. Total hip arthroplasty versus resurfacing arthroplasty in the treatment of patients with arthritis of the hip joint: single centre, parallel group, assessor blinded, randomised controlled trial. BMJ. 2012;344:e2147.
29. Costa ML, Achten J, Parsons NR, Rangan A, Griffin D, Tubeuf S, et al. Percutaneous fixation with Kirschner wires versus volar locking plate fixation in adults with dorsally displaced fracture of distal radius: randomised controlled trial. BMJ. 2014;349:g4807.
30. McCulloch P. Developing appropriate methodology for the study of surgical techniques. Journal of the Royal Society of Medicine. 2009;102:51-5.
31. Achten J, Parsons NR, Bruce J, Petrou S, Tutton E, Willett K, et al. Protocol for a randomised controlled trial of standard wound management versus negative pressure wound therapy in the treatment of adult patients with an open fracture of the lower limb: UK Wound management of Open Lower Limb Fractures (UK WOLFF). BMJ Open. 2015;5(9):e009087.
32. Bhatt DL, Mehta C. Adaptive Designs for Clinical Trials. N Engl J Med. 2016;375(1):65-74.
33. U.S. Department of Health and Human Services Food and Drug Administration. Adaptive Designs for Medical Device Clinical Studies final guidance2016 21/04/2017. Available from:

For peer review only - http://bmjopen.bmj.com/site/about/guidelines.xhtml

https://www.fda.gov/downloads/medicaldevices/deviceregulationandguidance/guidancedocuments/ucm446729.pdf.
34. Dimairo M, Boote J, Julious SA, Nicholl JP, Todd S. Missing steps in a staircase: a qualitative study of the perspectives of key stakeholders on the use of adaptive designs in confirmatory trials. Trials. 2015;16:430.
35. Campbell G, Yue LQ. Statistical innovations in the medical device world sparked by the FDA. J Biopharm Stat. 2016;26(1):3-16.
36. Elsasser A, Regnstrom J, Vetter T, Koenig F, Hemmings RJ, Greco M, et al. Adaptive clinical trial designs for European marketing authorization: a survey of scientific advice letters from the European Medicines Agency. Trials. 2014;15:383.
37. Kairalla Ja, Coffey CS, Thomann Ma, Muller KE. Adaptive trial designs: a review of barriers and opportunities. Trials. 2012;13:145.
38. Parsons N, Stallard N, Parsons H, Wells P, Underwood M, Mason J, et al. An adaptive two-arm clinical trial using early endpoints to inform decision making: design for a study of sub-acromial spacers for repair of rotator cuff tendon tear Trials. 2019;(in press).
39. Constant CR, Gerber C, Emery RJ, Sojbjerg JO, Gohlke F, Boileau P. A review of the Constant score: modifications and guidelines for its use. J Shoulder Elbow Surg. 2008;17(2):355-61.
40. Constant CR, Murley AH. A clinical method of functional assessment of the shoulder. Clin Orthop Relat Res. 1987(214):160-4.
41. Moeller AD, Thorsen RR, Torabi TP, Bjoerkman AS, Christensen EH, Maribo T, et al. The Danish version of the modified Constant-Murley shoulder score: reliability, agreement, and construct validity. J Orthop Sports Phys Ther. 2014;44(5):336-40.
42. Ban I, Troelsen A, Christiansen DH, Svendsen SW, Kristensen MT. Standardised test protocol (Constant Score) for evaluation of functionality in patients with shoulder disorders. Dan Med J. 2013;60(4):A4608.
43. Blonna D, Scelsi M, Marini E, Bellato E, Tellini A, Rossi R, et al. Can we improve the reliability of the Constant-Murley score? J Shoulder Elbow Surg. 2012;21(1):4-12.
44. Christiansen DH, Frost P, Falla D, Haahr JP, Frich LH, Svendsen SW. Responsiveness and Minimal Clinically Important Change: A Comparison Between 2 Shoulder Outcome Measures. J Orthop Sports Phys Ther. 2015;45(8):620-5.
45. Roy JS, MacDermid JC, Woodhouse LJ. A systematic review of the psychometric properties of the Constant-Murley score. J Shoulder Elbow Surg. 2010;19(1):157-64.
46. Khatri C, Ahmed I, Parsons H, Smith NA, Lawrence TM, Modi CS, et al. The Natural History of Full-Thickness Rotator Cuff Tears in Randomized Controlled Trials: A Systematic Review and Meta-analysis. The American journal of sports medicine. 2019;47(7):1734-43.
47. Dawson J, Rogers K, Fitzpatrick R, Carr A. The Oxford shoulder score revisited. Archives of Orthopaedic and Trauma Surgery. 2009;129(1):119-23.
48. Dawson J, Fitzpatrick R, Carr A. Questionnaire on the perceptions of patients about shoulder surgery. J Bone Joint Surg Br. 1996;78(4):593-600.
49. Holtby R, Razmjou H. Measurement properties of the Western Ontario rotator cuff outcome measure: a preliminary report. J Shoulder Elbow Surg. 2005;14(5):506-10.
50. Brooks R. EuroQol: the current state of play. Health Policy. 1996;37(1):53-72.
51. Janssen MF, Pickard AS, Golicki D, Gudex C, Niewada M, Scalone L, et al. Measurement properties of the EQ-5D-5L compared to the EQ-5D-3L across eight patient groups: a multi-country study. Qual Life Res. 2013;22(7):1717-27.

For peer review only - http://bmjopen.bmj.com/site/about/guidelines.xhtml

52. Kamper SJ, Maher CG, Mackay G. Global rating of change scales: a review of strengths and weaknesses and considerations for design. J Man Manip Ther. 2009;17(3):163-70.
53. Kellgren JH, Lawrence JS. Radiological assessment of osteo-arthrosis. Ann Rheum Dis. 1957;16(4):494-502.
54. Burkhart SS. Reconciling the paradox of rotator cuff repair versus debridement: a unified biomechanical rationale for the treatment of rotator cuff tears. Arthroscopy. 1994;10(1):4-19.
55. Huegel J, Williams AA, Soslowsky LJ. Rotator cuff biology and biomechanics: a review of normal and pathological conditions. Current rheumatology reports. 2015;17:476.
56. Gumina S, Arceri V, Fagnani C, Venditto T, Catalano C, Candela V, et al. Subacromial Space Width: Does Overuse or Genetics Play a Greater Role in Determining It? An MRI Study on Elderly Twins. J Bone Joint Surg Am. 2015;97(20):1647-52.
57. McCreesh KM, Crotty JM, Lewis JS. Acromiohumeral distance measurement in rotator cuff tendinopathy: is there a reliable, clinically applicable method? A systematic review. Br J Sports Med. 2015;49(5):298-305.
58. Tempelaere C, Pierrart J, Lefevre-Colau MM, Vuillemin V, Cuenod CA, Hansen U, et al. Dynamic Three-Dimensional Shoulder Mri during Active Motion for Investigation of Rotator Cuff Diseases. PLoS One. 2016;11(7):e0158563.
59. Haahr JP, Ostergaard S, Dalsgaard J, Norup K, Frost P, Lausen S, et al. Exercises versus arthroscopic decompression in patients with subacromial impingement: a randomised, controlled study in 90 cases with a one year follow up. Ann Rheum Dis. 2005;64(5):760-4.
60. Karthikeyan S, Kwong HT, Upadhyay PK, Parsons N, Drew SJ, Griffin D. A double-blind randomised controlled study comparing subacromial injection of tenoxicam or methylprednisolone in patients with subacromial impingement. J Bone Joint Surg Br. 2010;92(1):77-82.
61. Kalra N, Seitz AL, Boardman ND, 3rd, Michener LA. Effect of posture on acromiohumeral distance with arm elevation in subjects with and without rotator cuff disease using ultrasonography. J Orthop Sports Phys Ther. 2010;40(10):633-40.
62. Moher D, Schulz KF, Altman DG. The CONSORT statement: revised recommendations for improving the quality of reports of parallel-group randomised trials. The Lancet. 2001;357:1191-4.
63. Dimairo M, Coates E, Pallmann P, Todd S, Julious SA, Jaki T, et al. Development process of a consensus-driven CONSORT extension for randomised trials using an adaptive design. BMC Med. 2018;16(1):210.
64. Whitehead J. Overrunning and underrunning in sequential clinical trials. Control Clin Trials. 1992;13(2):106-21.
65. Liu A, Hall WJ. Unbiased estimation following a group sequential test. . Biometrika. 1999;86:71-8.
66. Todd S, Whitehead J, Facey KM. Point and interval estimation following a sequential clinical trial. . Biometrika. 1996; 83:453-61.
67. Pincus T, Miles C, Froud R, Underwood M, Carnes D, Taylor SJ. Methodological criteria for the assessment of moderators in systematic reviews of randomised controlled trials: a consensus study. BMC Med Res Methodol. 2011;11:14.
68. National Institute for Health and Care Excellence. Guide to the methods of technology appraisal 2013. Process and methods [PMG9] [Internet]. 2013 11/04/17.

For peer review only - http://bmjopen.bmj.com/site/about/guidelines.xhtml

Available from: https://www.nice.org.uk/process/pmg9/resources/guide-to-the-methods-of-technology-appraisal-2013-pdf-2007975843781.
69. Manca A, Hawkins N, Sculpher MJ. Estimating mean QALYs in trial-based cost-effectiveness analysis: the importance of controlling for baseline utility. Health Econ. 2005;14(5):487-96.
70. Sterne JA, White IR, Carlin JB, Spratt M, Royston P, Kenward MG, et al. Multiple imputation for missing data in epidemiological and clinical research: potential and pitfalls. BMJ. 2009;338:b2393.
71. White IR, Horton NJ, Carpenter J, Pocock SJ. Strategy for intention to treat analysis in randomised trials with missing outcome data. BMJ. 2011;342:d40.
72. White IR, Royston P, Wood AM. Multiple imputation using chained equations: Issues and guidance for practice. Stat Med. 2011;30(4):377-99.
73. Chen MH, Willan AR. Determining optimal sample sizes for multistage adaptive randomized clinical trials from an industry perspective using value of information methods. Clin Trials. 2013;10(1):54-62.
74. Willan A, Kowgier M. Determining optimal sample sizes for multi-stage randomized clinical trials using value of information methods. Clin Trials. 2008;5(4):289-300.

For peer review only

For peer review only - http://bmjopen.bmj.com/site/about/guidelines.xhtml

Reporting checklist for protocol of a clinical trial.

Based on the SPIRIT guidelines.

Instructions to authors

Complete this checklist by entering the page numbers from your manuscript where readers will find each of the items listed below.

Your article may not currently address all the items on the checklist. Please modify your text to include the missing information. If you are certain that an item does not apply, please write "n/a" and provide a short explanation.

Upload your completed checklist as an extra file when you submit to a journal.

In your methods section, say that you used the SPIRITreporting guidelines, and cite them as:

Chan A-W, Tetzlaff JM, Altman DG, Laupacis A, Gøtzsche PC, Krleža-Jerić K, Hróbjartsson A, Mann H, Dickersin K, Berlin J, Doré C, Parulekar W, Summerskill W, Groves T, Schulz K, Sox H, Rockhold FW, Rennie D, Moher D. SPIRIT 2013 Statement: Defining standard protocol items for clinical trials. Ann Intern Med. 2013;158(3):200-207

		Reporting Item	Page Number
**Administrative information**			
Title	#1	Descriptive title identifying the study design, population, interventions, and, if applicable, trial acronym	1
Trial registration	#2a	Trial identifier and registry name. If not yet registered, name of intended registry	2
Trial registration: data set	#2b	All items from the World Health Organization Trial Registration Data Set	2
Protocol version	#3	Date and version identifier	n/a
Funding	#4	Sources and types of financial, material, and other support	16
Roles and responsibilities: contributorship	#5a	Names, affiliations, and roles of protocol contributors	1,16

For peer review only

Roles and responsibilities: sponsor contact information	#5b	Name and contact information for the trial sponsor	16
Roles and responsibilities: sponsor and funder	#5c	Role of study sponsor and funders, if any, in study design; collection, management, analysis, and interpretation of data; writing of the report; and the decision to submit the report for publication, including whether they will have ultimate authority over any of these activities	16
Roles and responsibilities: committees	#5d	Composition, roles, and responsibilities of the coordinating centre, steering committee, endpoint adjudication committee, data management team, and other individuals or groups overseeing the trial, if applicable (see Item 21a for data monitoring committee)	6,7
**Introduction**			
Background and rationale	#6a	Description of research question and justification for undertaking the trial, including summary of relevant studies (published and unpublished) examining benefits and harms for each intervention	4,5
Background and rationale: choice of comparators	#6b	Explanation for choice of comparators	4,5
Objectives	#7	Specific objectives or hypotheses	6
Trial design	#8	Description of trial design including type of trial (eg, parallel group, crossover, factorial, single group), allocation ratio, and framework (eg, superiority, equivalence, non-inferiority, exploratory)	6
**Methods: Participants, interventions, and outcomes**			
Study setting	#9	Description of study settings (eg, community clinic, academic hospital) and list of countries where data will be	6,9

For peer review only

		collected. Reference to where list of study sites can be obtained	
Eligibility criteria	#10	Inclusion and exclusion criteria for participants. If applicable, eligibility criteria for study centres and individuals who will perform the interventions (eg, surgeons, psychotherapists)	8,9
Interventions: description	#11a	Interventions for each group with sufficient detail to allow replication, including how and when they will be administered	10
Interventions: modifications	#11b	Criteria for discontinuing or modifying allocated interventions for a given trial participant (eg, drug dose change in response to harms, participant request, or improving / worsening disease)	n/a
Interventions: adherance	#11c	Strategies to improve adherence to intervention protocols, and any procedures for monitoring adherence (eg, drug tablet return; laboratory tests)	10
Interventions: concomitant care	#11d	Relevant concomitant care and interventions that are permitted or prohibited during the trial	10
Outcomes	#12	Primary, secondary, and other outcomes, including the specific measurement variable (eg, systolic blood pressure), analysis metric (eg, change from baseline, final value, time to event), method of aggregation (eg, median, proportion), and time point for each outcome. Explanation of the clinical relevance of chosen efficacy and harm outcomes is strongly recommended	7
Participant timeline	#13	Time schedule of enrolment, interventions (including any run-ins and washouts), assessments, and visits for participants. A schematic diagram is highly recommended (see Figure)	6,7,9,11
Sample size	#14	Estimated number of participants needed to achieve study objectives and how it was determined, including clinical and statistical assumptions supporting any sample size calculations	12,13
Recruitment	#15	Strategies for achieving adequate participant enrolment to reach target sample size	9

For peer review only

**Methods: Assignment of interventions (for controlled trials)**			
Allocation: sequence generation	#16a	Method of generating the allocation sequence (eg, computer-generated random numbers), and list of any factors for stratification. To reduce predictability of a random sequence, details of any planned restriction (eg, blocking) should be provided in a separate document that is unavailable to those who enrol participants or assign interventions	9
Allocation concealment mechanism	#16b	Mechanism of implementing the allocation sequence (eg, central telephone; sequentially numbered, opaque, sealed envelopes), describing any steps to conceal the sequence until interventions are assigned	9
Allocation: implementation	#16c	Who will generate the allocation sequence, who will enrol participants, and who will assign participants to interventions	9,10,11
Blinding (masking)	#17a	Who will be blinded after assignment to interventions (eg, trial participants, care providers, outcome assessors, data analysts), and how	10,11
Blinding (masking): emergency unblinding	#17b	If blinded, circumstances under which unblinding is permissible, and procedure for revealing a participant's allocated intervention during the trial	11
**Methods: Data collection, management, and analysis**			
Data collection plan	#18a	Plans for assessment and collection of outcome, baseline, and other trial data, including any related processes to promote data quality (eg, duplicate measurements, training of assessors) and a description of study instruments (eg, questionnaires, laboratory tests) along with their reliability and validity, if known. Reference to where data collection forms can be found, if not in the protocol	7,8,9,10,11,12

For peer review only

Data collection plan: retention	#18b	Plans to promote participant retention and complete follow-up, including list of any outcome data to be collected for participants who discontinue or deviate from intervention protocols	7,12
Data management	#19	Plans for data entry, coding, security, and storage, including any related processes to promote data quality (eg, double data entry; range checks for data values). Reference to where details of data management procedures can be found, if not in the protocol	8
Statistics: outcomes	#20a	Statistical methods for analysing primary and secondary outcomes. Reference to where other details of the statistical analysis plan can be found, if not in the protocol	13,14,15
Statistics: additional analyses	#20b	Methods for any additional analyses (eg, subgroup and adjusted analyses)	13,14,15
Statistics: analysis population and missing data	#20c	Definition of analysis population relating to protocol non-adherence (eg, as randomised analysis), and any statistical methods to handle missing data (eg, multiple imputation)	13,14,15
**Methods: Monitoring**			
Data monitoring: formal committee	#21a	Composition of data monitoring committee (DMC); summary of its role and reporting structure; statement of whether it is independent from the sponsor and competing interests; and reference to where further details about its charter can be found, if not in the protocol. Alternatively, an explanation of why a DMC is not needed	6,7,11,13,14
Data monitoring: interim analysis	#21b	Description of any interim analyses and stopping guidelines, including who will have access to these interim results and make the final decision to terminate the trial	11,14
Harms	#22	Plans for collecting, assessing, reporting, and managing solicited and spontaneously reported adverse events and other unintended effects of trial interventions or trial conduct	12
Auditing	#23	Frequency and procedures for auditing trial conduct, if any, and whether the process will be independent from investigators and the sponsor	7

For peer review only

Ethics and dissemination

Research ethics approval	#24	Plans for seeking research ethics committee / institutional review board (REC / IRB) approval	7
Protocol amendments	#25	Plans for communicating important protocol modifications (eg, changes to eligibility criteria, outcomes, analyses) to relevant parties (eg, investigators, REC / IRBs, trial participants, trial registries, journals, regulators)	7
Consent or assent	#26a	Who will obtain informed consent or assent from potential trial participants or authorised surrogates, and how (see Item 32)	9
Consent or assent: ancillary studies	#26b	Additional consent provisions for collection and use of participant data and biological specimens in ancillary studies, if applicable	n/a
Confidentiality	#27	How personal information about potential and enrolled participants will be collected, shared, and maintained in order to protect confidentiality before, during, and after the trial	7,8,9,11,12
Declaration of interests	#28	Financial and other competing interests for principal investigators for the overall trial and each study site	16
Data access	#29	Statement of who will have access to the final trial dataset, and disclosure of contractual agreements that limit such access for investigators	13
Ancillary and post trial care	#30	Provisions, if any, for ancillary and post-trial care, and for compensation to those who suffer harm from trial participation	n/a
Dissemination policy: trial results	#31a	Plans for investigators and sponsor to communicate trial results to participants, healthcare professionals, the public, and other relevant groups (eg, via publication, reporting in results databases, or other data sharing arrangements), including any publication restrictions	15
Dissemination policy: authorship	#31b	Authorship eligibility guidelines and any intended use of professional writers	15

For peer review only

Dissemination policy: reproducible research	#31c	Plans, if any, for granting public access to the full protocol, participant-level dataset, and statistical code	n/a

Appendices

Informed consent materials	#32	Model consent form and other related documentation given to participants and authorised surrogates	n/a
Biological specimens	#33	Plans for collection, laboratory evaluation, and storage of biological specimens for genetic or molecular analysis in the current trial and for future use in ancillary studies, if applicable	n/a

Notes:

- 18a: 7,8,9,10,11,12
- 21a: 6,7,11,13,14
- 27: 7,8,9,11,12 The SPIRIT checklist is distributed under the terms of the Creative Commons Attribution License CC-BY-ND 3.0. This checklist was completed on 03. January 2020 using https://www.goodreports.org/, a tool made by the EQUATOR Network in collaboration with Penelope.ai

For peer review only

BMJ Open

Protocol for a randomised controlled trial of Subacromial spacers for Tears Affecting Rotator cuff Tendons: a Randomised, Efficient, Adaptive Clinical Trial in Surgery (START:REACTS)

Journal:	*BMJ Open*
Manuscript ID	bmjopen-2020-036829.R1
Article Type:	Protocol
Date Submitted by the Author:	01-Apr-2020
Complete List of Authors:	Metcalfe, Andrew; University of Warwick Warwick Medical School, Warwick Clinical Trials Unit; University Hospitals Coventry and Warwickshire NHS Trust, Trauma and Orthopaedics<br>Gemperle Mannion, Elke; University of Warwick Warwick Medical School, Warwick Clinical Trials Unit<br>Parsons, Helen; University of Warwick, Division of Health Sciences<br>Brown, Jaclyn; University of Warwick, Clinical Trials Unit; University of Warwick Clinical Sciences Research Institute, CTU<br>Parsons, Nicholas; University of Warwick, Warwick Medical School<br>Fox, Jo; Patient representative<br>Kearney, Rebecca; University of Warwick, Warwick Clinical Trials Unit<br>Lawrence, Tom; University Hospitals Coventry and Warwickshire NHS Trust, Trauma and Orthopaedics<br>Bush, Howard; University Hospitals Coventry and Warwickshire NHS Trust, Trauma and Orthopaedics<br>McGowan, Kerri; University Hospitals Coventry and Warwickshire NHS Trust<br>Khan, Iftekhar; University of Warwick Warwick Medical School, Centre for Health Economics at Warwick<br>Mason, James; University of Warwick, Warwick Medical School<br>Hutchinson, Charles; University of Warwick, Warwick Medical School<br>Gates, Simon; University of Birmingham, Cancer Research UK Clinical Trials Unit; University of Warwick, Clinical Trials Unit<br>Stallard, Nigel; University of Warwick,<br>Underwood, Martin; Warwick University, Warwick Medical School<br>Drew, Stephen; University Hospitals Coventry and Warwickshire NHS Trust, Trauma and Orthopaedics
**Primary Subject Heading**:	Surgery
Secondary Subject Heading:	Research methods, Health economics, Evidence based practice
Keywords:	Shoulder < ORTHOPAEDIC & TRAUMA SURGERY, Adult orthopaedics < ORTHOPAEDIC & TRAUMA SURGERY, Clinical trials < THERAPEUTICS

SCHOLARONE™
Manuscripts

I, the Submitting Author has the right to grant and does grant on behalf of all authors of the Work (as defined in the below author licence), an exclusive licence and/or a non-exclusive licence for contributions from authors who are: i) UK Crown employees; ii) where BMJ has agreed a CC-BY licence shall apply, and/or iii) in accordance with the terms applicable for US Federal Government officers or employees acting as part of their official duties; on a worldwide, perpetual, irrevocable, royalty-free basis to BMJ Publishing Group Ltd ("BMJ") its licensees and where the relevant Journal is co-owned by BMJ to the co-owners of the Journal, to publish the Work in this journal and any other BMJ products and to exploit all rights, as set out in our licence.

The Submitting Author accepts and understands that any supply made under these terms is made by BMJ to the Submitting Author unless you are acting as an employee on behalf of your employer or a postgraduate student of an affiliated institution which is paying any applicable article publishing charge ("APC") for Open Access articles. Where the Submitting Author wishes to make the Work available on an Open Access basis (and intends to pay the relevant APC), the terms of reuse of such Open Access shall be governed by a Creative Commons licence – details of these licences and which Creative Commons licence will apply to this Work are set out in our licence referred to above.

Other than as permitted in any relevant BMJ Author's Self Archiving Policies, I confirm this Work has not been accepted for publication elsewhere, is not being considered for publication elsewhere and does not duplicate material already published. I confirm all authors consent to publication of this Work and authorise the granting of this licence.

For peer review only

Protocol for a randomised controlled trial of Subacromial spacers for Tears Affecting Rotator cuff Tendons: a Randomised, Efficient, Adaptive Clinical Trial in Surgery (START:REACTS)

Authors:

Mr Andrew Metcalfe[1,2] <a.metcalfe@warwick.ac.uk> (corresponding author)

Mrs Elke Gemperle Mannion[2] <E.Gemperle-Mannion@warwick.ac.uk>

Dr Helen Parsons[2] <H.Parsons@warwick.ac.uk>

Ms Jaclyn Brown[2] <J.Brown.10@warwick.ac.uk>

Dr Nicholas Parsons[3] <nick.parsons@warwick.ac.uk>

Miss Jo Fox[4] <josephine@bonesmart.org>

Dr Rebecca Kearney[1,2] <R.S.Kearney@warwick.ac.uk>

Mr Tom Lawrence[1] <Tom.Lawrence@uhcw.nhs.uk>

Mr Howard Bush[1] <Howard.Bush@uhcw.nhs.uk>

Mrs Kerri McGowan[1] <kerri.mcgowan@uhcw.nhs.uk>

Dr Iftekhar Khan[3] <I.Khan.2@warwick.ac.uk>

Professor James Mason[2] <J.Mason@warwick.ac.uk>

Professor Charles Hutchinson[3] <C.E.Hutchinson@warwick.ac.uk>

Professor Simon Gates[5] <S.Gates@bham.ac.uk>

Professor Nigel Stallard[3] <N.Stallard@warwick.ac.uk>

Professor Martin Underwood[1,2] <M.Underwood@warwick.ac.uk>

Mr Stephen Drew[1] <Steven.Drew@uhcw.nhs.uk>

1. University Hospitals Coventry and Warwickshire NHS Trust, Coventry, UK
2. Warwick Clinical Trials Unit, University of Warwick, UK
3. Warwick Medical School, University of Warwick, UK
4. Patient member
5. University of Birmingham Clinical Trials Unit

For peer review only

For peer review only - http://bmjopen.bmj.com/site/about/guidelines.xhtml

Abstract

Introduction

Shoulder pain due to irreparable rotator cuff tears can cause substantial disability, but treatment options are limited. A balloon spacer is a relatively simple addition to a standard arthroscopic debridement procedure, but it is costly and there is no current randomised trial evidence to support its use. This trial will evaluate the clinical and cost effectiveness of a subacromial balloon spacer for individuals undergoing arthroscopic debridement for irreparable rotator cuff tears.

New surgical procedures can provide substantial benefit to patients. Good quality randomised controlled trials (RCTs) are needed, but trials in surgery are typically long and expensive, exposing patients to risk and the healthcare system to substantial costs. One way to improve the efficiency of trials is with an adaptive sample size. Such methods are well established in drug trials but have rarely, if ever, been used in surgical trials.

Methods and analysis

START:REACTS is a participant and assessor blinded, adaptive, multi-centre RCT comparing arthroscopic debridement with the InSpace™ balloon (Stryker, USA) to arthroscopic debridement alone for people with a symptomatic irreparable rotator cuff tear. It uses a group sequential adaptive design where interim analyses are performed utilising all of the three, six and twelve month data that are available at each time point. A maximum of 221 participants will be randomised (1:1 ratio), this will provide 90% power (at the 5% level) for a 6 point difference in the primary outcome; the Oxford Shoulder Score at 12 months. A sub-study will use deltoid-active MRI scans in 56 participants to assess the function of the balloon. Analysis will be on an intention-to-treat basis and reported according to principles established in the CONSORT statement.

Ethics and dissemination

NRES number 18/WM/0025. The results will be disseminated via peer-reviewed publications, presentations at conferences, lay summaries and social media.

Trial Registration number: ISRCTN17825590.

For peer review only

For peer review only - http://bmjopen.bmj.com/site/about/guidelines.xhtml

Keywords

Randomised Controlled Trial; Shoulder; Rotator Cuff; Subacromial spacer balloon; adaptive design;

Article Summary

Strengths and limitations of this study

- Multi-centre randomised trial of a subacromial spacer balloon following debridement, compared to debridement alone, for irreparable rotator cuff tears of the shoulder.
- Participant-assessor blinding, including blinded operation notes with novel unblinding mechanism.
- Mechanistic MRI sub-study of 56 participants with images at eight weeks and six months after surgery.
- Statistical adaptive design, with hard stopping rules for futility or efficacy based on emerging outcomes.

For peer review only - http://bmjopen.bmj.com/site/about/guidelines.xhtml

INTRODUCTION

Subacromial spacer balloons

Shoulder pain is a common and disabling problem. The UK population prevalence of shoulder pain is approximately 16%.(1) Rotator cuff disease accounts for 70% - 85% of this.(2-4) People with a symptomatic rotator cuff tear typically have pain, restricted movement, loss of strength and disability. The condition is associated with substantial expense to society through both costs of treatment and loss of work (both paid and unpaid).(5-8) Rotator cuff repair is a widely accepted treatment for symptomatic rotator cuff tears.(9, 10) Some tears cannot be surgically repaired, these are called irreparable tears.

Symptomatic irreparable rotator cuff tears are a challenging condition to treat. Treatment options including physiotherapy, injections, arthroscopic debridement, partial repair, muscle transfers, interposition grafts and even shoulder replacements.(11-14) Arthroscopic debridement is commonly used and case-series suggest it may be beneficial, but it remains a controversial option, with little randomised controlled trial data on its use in the irreparable tear population.(15-17)

In 2013**,** the InSpace subacromial balloon spacer (Stryker, Michigan, USA) was introduced into UK orthopaedic practice as a potential treatment option for people with irreparable tears of the rotator cuff. In May 2016 an interventional procedure guidance document was published by the UK National Institute for Health and Care Excellence (NICE), which found that there was very limited evidence for its use. Therefore, the device was limited to research use only and a recommendation was made to assess its effectiveness.(18) It has not yet received U.S. Food and Drug Administration clearance in the United States of America, but it is a widely used treatment elsewhere in the world.

The InSpace device is a saline-filled balloon made of biodegradable (dissolvable) synthetic material. It is inserted above the main joint of the shoulder at the end of an arthroscopic debridement after an irreparable tear has been identified. It is simple to deploy and adds relatively little time to the operation.(19, 20) It cushions the humerus from pressing on the bone above it (the acromion) when the deltoid is active and during abduction of the arm, potentially reducing pain. It may also assist in the biomechanics of the shoulder, resisting proximal migration of the humerus under deltoid activity. The device begins to degrade and deflate from three months after surgery. During this time, it is thought to improve rehabilitation of the remaining rotator cuff and deltoid, so that when the device deflates, the biomechanics of the shoulder are better preserved.

The safety of the device in rodents has been established, with only one adverse event: a fibrosarcoma that was thought to be unique to rodents.(21) Proof of concept has been established in a series of 24 irreparable cuff tear cases in Slovakia in 2012, with 5-year follow-up results published in 2016.(22, 23) The device has been used in a number of centres across the UK, with multiple conference abstracts from the UK.(24-26) These have demonstrated improvements in outcomes from baseline. Complications such as balloon displacement and non-cyst forming synovitis have been reported in a small number of cases (3 out of 61). One retrospective non-randomised study of 23 patients (12 with the balloon)

For peer review only - http://bmjopen.bmj.com/site/about/guidelines.xhtml

showed an improvement in outcomes compared to debridement alone.(27) There are no completed randomized controlled trials (RCTs).

The benefits found in case series may not be unique to the InSpace balloon, although the relative effectiveness of the balloon in comparison to non-operative care or acromioplasty is not known and may give substantial improvements.(17)

There is one other RCT in progress for this device – a company funded study in the USA which will recruit 184 participants, comparing partial cuff repair with balloon as a stand-alone intervention (clinicaltrials.gov NCT02493660). Results are expected in mid-2020. Partial cuff repair is not a technique that is widely used in the UK and is not an appropriate comparator in a UK context.

Trial designs in new surgical procedures

The safe introduction of new surgical procedures is essential to the delivery of high quality surgical care for patients. New procedures can result in a step-change improvement in treatment, but also introduce new risks, and substantial costs. Major harm can be done when a misunderstood or well-intentioned intervention is used widely across the health service before it is formally evaluated. (28, 29)

While pharmaceuticals must undergo rigorous clinical trials before introduction, this is not the case for surgical procedures.(30) When surgical procedures are assessed rigorously, studies tend to be large multi-centre, randomised trials which typically need to recruit over extended time periods.(29, 31) Improvements in the efficiency of delivering trials of surgical interventions would provide earlier answers to these important clinical questions, providing benefits to patients and making better use of healthcare resources.

The use of adaptive trial designs has been encouraged by major journals, the US Food and Drug Administration, and National Institute of Health Research (NIHR) panels.(32-34) Adaptive trial designs allow prospectively planned modifications, such as stopping the study early or dropping an intervention, based on emerging findings as the trial proceeds, while preserving the scientific validity and integrity of the trial. This flexible strategy typically reduces costs and shortens time-scales, without compromising the integrity, statistical power or rigour of the study.(32, 35-37)

In this trial we are using a novel statistical adaptive design approach for the assessment of a new surgical procedure. The statistical principles are laid out in a 2019 methodology paper.(38) Briefly, the design uses early (three and six month Oxford Shoulder Score) endpoint data to support emerging outcome data for the primary endpoint (12 month Oxford Shoulder Score) to make decisions about stopping for either futility or efficacy at pre-planned interim analyses that occur before the end of recruitment. In the current study, we will apply these design principles to a multi-centre clinical trial of a new surgical procedure.

Aim

To assess the clinical-effectiveness, cost-effectiveness and safety of a sub-acromial spacer balloon for patients with symptomatic irreparable tears of the rotator cuff.(18)

For peer review only - http://bmjopen.bmj.com/site/about/guidelines.xhtml

METHODS

Trial Design

The Subacromial spacer for Tears Affecting Rotator cuff Tendons (START) study is a participant and assessor blinded, adaptive, multi-centre RCT based in the UK comparing arthroscopic debridement using the InSpace balloon to arthroscopic debridement alone, performed using the REACTS (Randomised, Efficient, Adaptive Clinical Trial in Surgery) framework.

Patient and Public Involvement

Patient involvement has been a core part of the design and delivery of the study, and will remain so, including in the interpretation and dissemination of results. In the planning stages, we engaged with multiple patients who had previously undergone rotator cuff surgery and their insights helped establish the design of the study, especially the outcomes. We reviewed patient facing materials with many shoulder patients before they wre finalised. One of the co-authors is a patient and represents the patient view in trial management meetings, two patients sit on our steering committee. We will produce patient and public-focused summaries of the research and disseminate this widely.

Objectives

Clinical objectives

- Our **primary clinical objective** is to quantify and draw inferences on observed differences between arthroscopic debridement of the subacromial space and arthroscopic debridement with insertion of the InSpace balloon (Stryker, Michigan, USA) 12 months after surgery, using the Oxford Shoulder Score as the primary outcome measure.(39, 40)

Secondary clinical objectives:

- To quantify and draw inferences on observed differences between arthroscopic debridement and arthroscopic debridement with insertion of the InSpace balloon (Stryker, Michigan, USA) on: Shoulder function; patient reported outcome measures; adverse events and; resource use at three, six and 12 months.

- To perform an economic analysis, assessing the comparative cost-effectiveness of the two treatments.

- To compare the acromio-humeral distance on MRI scans in a sample of participants with and without the balloon at eight weeks and six months after treatment, to assess the proposed mechanism of action of the balloon when it is still inflated (at eight weeks) and to determine if the effect persists when it has deflated (at six months).

For peer review only - http://bmjopen.bmj.com/site/about/guidelines.xhtml

Methodological Objectives

- The **primary methodological objective** is to develop and implement appropriate statistical tools to allow an efficient adaptive clinical trial design, with the potential for early stopping for either futility or efficacy, using Oxford Shoulder Score data available at three, six and 12 months.(38)

Ethics, registration and oversight

The trial will conform to the principles of the Declaration of Helsinki and to MRC Good Clinical Practice (GCP) guidelines. It will also comply with all applicable UK legislation and University of Warwick Standard Operating Procedures (SOPs). Trial oversight is provided by a Data Monitoring Committee (DMC) and Trial Steering Committee (TSC), both are made up of a majority of independent members and are conducted according to Warwick SOPs. Monitoring and audit will be undertaken by the sponsors according to a monitoring plan. The trial received full research ethical approval (RES number 18/WM/0025) on 13th February 2018 and was registered on ISRCTN on the 6th April 2018 (ISRCTN17825590), prior to commencing recruitment which is ongoing. Amendments will be communicated to sites by the co-ordinating team.

Outcome measures

Primary outcome: The Oxford Shoulder Score at 12 months. The original study design was based around the Constant score, however the trial management group decided in March 2020, in light of the coronavirus outbreak, to revise this to the Oxford Shoulder Score. As the Constant score is a face-to-face measure usually taken in hospital clinics, it would have exposed participants to unnecessary risk during the height of the pandemic. The decision was agreed by both the Trial Steering committees and Data Monitoring Committees prior to the change.

The Oxford Shoulder Score has been well validated and used in high-impact randomised trials previously, it correlates well with the Constant score, both are similarly responsive and have comparable effect sizes in rotator cuff pathology.(39-43) Based on our meta-analysis of outcomes for randomised trials, the shoulder scores typically reach a plateau at 12 months after any intervention for a rotator cuff tear.(44) The 24 month scores do not give sufficient additional value to justify the increase in costs and delay in the trial result that would be required had 24 months been used as the primary outcome.

Secondary outcomes:

- The Oxford Shoulder Score (OSS) at baseline, three, six, and 24 months.
- The Constant Score at baseline, three, and six and 12 months. A standardised protocol for the objective component of the score has been developed based on the work of Moeller *et al.* with training provided for all sites. (45-47)

For peer review only - http://bmjopen.bmj.com/site/about/guidelines.xhtml

- Range of pain-free movement of the shoulder at baseline, three, six and 12 months measured using a long-arm goniometer (12 ½”).
- Strength of shoulder abduction and flexion at baseline, three, six, and 12 months measured using a supplied IsoForceControl EVO2 dynamometer (Herkules Kunstoff, Switzerland).
- Western Ontario Rotator Cuff index at baseline, three, six, 12 and 24 months.(48)
- Health utility assessed using EQ-5D-5L at baseline, three, six , 12 and 24 months.(49, 50)
- Healthcare resource collected at three, six, and 12 months
- Patient global assessment of change (PGIC) score, collected at three, six, 12 and 24 months. A simple seven-point scale assessing perception of improvement.(51)
- Analgesia use (drug and approximate frequency) collected at baseline and three, six and 12 months.
- MRI Scans (sub-study of 56 patients, six weeks and six months post-surgery): see ‘MRI sub-study’ below.
- Adverse events will be collected from site reports as they occur throughout the first 12 months, and from participants in the 3, 6 and 12 months questionnaires.

Patient reported outcome measures (OSS, WORC, EQ-5D-5L, PGIC) will be collected at 24 months as a secondary outcome, these will be published separately and will not delay publication of the primary 12 month outcome data. A detailed data management plan has been prepared following Warwick SOPs and will be available on request.

Eligibility criteria

Inclusion criteria

1. Rotator cuff tear deemed by the treating clinician to be technically irreparable*. Many factors other than size influence whether a tear can be repaired (such as chronicity, retraction of the tendon ends, fat infiltration in muscle). However, a potential participant who has a tear that is technically repairable, such as a small tear, but is unsuitable for repair due to age or co-morbidities, is not eligible for this study.
2. Intrusive symptoms (pain and loss of function) which in the opinion of the treating clinician warrants surgery.
3. Non-operative management has been unsuccessful. The exact nature of non-operative management will be left for the treating clinician to decide.

For peer review only - http://bmjopen.bmj.com/site/about/guidelines.xhtml

Exclusion criteria

4. Advanced gleno-humeral osteoarthritis (OA) on pre-operative imaging (in the opinion of the treating clinician). Advanced gleno-humeral OA may be interpreted as Kellgren Lawrence grade 3 or 4 changes on routine pre-operative radiographs(52), or the MRI equivalent if radiographs have not been taken.

5. Subscapularis deficiency*, defined as a tear involving more than the superior 1cm (approximately) of the subscapularis if repaired, or any tear that is not repaired.

6. The treating clinician determines that interposition grafting or tendon transfers are indicated.

7. Pseudoparalysis (an inability to actively abduct or forward flex up to 20°), as determined by the treating clinician.

8. Unrelated, symptomatic ipsilateral shoulder disorder that would interfere with strength measurement or ability to perform rehabilitation

9. Other neurological or muscular condition that would interfere with strength measurement or ability to perform rehabilitation, in the opinion of the treating clinician.

10. Previous proximal humerus fracture that could influence shoulder function, as determined by the treating clinician.

11. Previous entry into the present trial (i.e. other shoulder).

12. Unable to complete trial procedures.

13. Age under 18

14. Unable to consent to the trial.

15. Unfit for surgery as defined by the treating clinician.

*criteria regarding whether the tear is technically repairable, and the integrity of the subscapularis are unreliably assessed by pre-operative imaging and will be reassessed in theatre, prior to randomisation. People not eligible to be enrolled in the trial will be treated according to the best judgement of the surgeon at the time.

Participant identification, screening and withdrawals

Potential participants will be identified by the attending clinical team by clinicians in intermediate or secondary care clinics, or from the surgical waiting list. The attending clinician will confirm appropriateness for study eligibility based on clinical assessment and standard care pre-operative imaging for that site.

For peer review only - http://bmjopen.bmj.com/site/about/guidelines.xhtml

All potential participants who meet the study entry criteria will be checked for eligibility and recorded on the monthly screening log. Potential participants who are willing to be approached by a suitably trained member of the research team will be provided with verbal and written information about the study, and will have the opportunity to discuss and ask questions about the study prior to informed consent being obtained.

Eligibility for the study is confirmed by the operating surgeon intra-operatively and patients may be excluded at this stage if the surgeon finds that the rotator cuff tear can be repaired. These excluded participants will be informed by letter that they are no longer taking part in the study. Baseline data will be retained to explore any differences between those who were deemed ineligible at surgery to those who were randomised.

Participants randomised into the study will be allowed to withdraw from follow-up at any time, without prejudice. This will have no effect on their current or future care.

Randomisation

Participants are randomly allocated on a 1:1 basis to the two treatment groups via a central computer-based randomisation system provided by Warwick Clinical Trials Unit (WCTU, independent of the study team). A minimisation algorithm is used to determine participant allocation, using site, gender, age group (<70 years and ≥70 years) and cuff tear size (as assessed by the operating surgeon, ≥3cm or <3cm) as factors, with a random element included to provide a 70% chance that the participant will receive the treatment that minimises the imbalance, to ensure unpredictability.

Randomisation will be performed by theatre staff, after the intra-operative findings have been checked (including cuff tear size) and eligibility is confirmed. Staff use an online system in a separate room to maintain blinding, with a 24 hour back-up automated telephone system available.

Trial treatment(s) / intervention

Standard Arthroscopic debridement (control): The control group will be an arthroscopic debridement of the subacromial space with removal of inflamed tissue (bursectomy) and unstable remnants of the torn tendon, limited bone resection of the acromion, retention of the coraco-acromial (CA) ligament, and biceps tenotomy (if not already torn). The anaesthetic will be left to the choice of the anaesthetist; this may include general or local anaesthesia. Surgeons may use their normal surgical technique, within the confines described in a trial specific surgical guideline (available at the trial website or at request from Warwick CTU).

Group 2 – Standard Arthroscopic debridement plus insertion of InSpace balloon (Intervention): Arthroscopic debridement, as described above, with insertion of the InSpace Balloon performed by sub-speciality trained shoulder surgeons. The same arthroscopic debridement will be performed as described for the control group. If allocated to the balloon procedure, the company's recommended surgical technique will be followed for sizing, insertion and deployment of the balloon, as documented in the surgical manual.

For peer review only - http://bmjopen.bmj.com/site/about/guidelines.xhtml

For both groups, fidelity will be assessed with an operative record form and arthroscopic photographs taken at the end of debridement and just before balloon inflation, and the number of physiotherapy visits for each participant will be documented in both arms.

Rehabilitation

Post-operative rehabilitation for both groups will be blind to treatment allocation and will include standardised post-operative information, home exercises and the offer of a minimum of three face-to-face physiotherapy appointments. Additional physiotherapy will be at the discretion of the trial sites. A physiotherapy trial manual will be followed to standardise rehabilitation progression. All materials were developed through a process of collecting current NHS protocols, manufacturer protocols, scoping the literature and expert consensus.

Blinding

Treatment allocation will be blinded for both patients and assessors, only the surgical teams at the time of the operation will be aware of the allocation. Theatre staff are asked not to discuss the balloon and to communicate the allocation by using methods such as holding up a piece of paper on which the allocation is clearly written. If participants are awake, drapes are used to obscure the participant's view and arthroscopic screens will be positioned in such a way that the patient is unable to see the procedure.

The incisions required for the two operations are similar and there is no external way in which the patients will be able to detect the presence or absence of the balloon, except for the size of the lateral portal. One of the incisions (the lateral portal) will need to be slightly larger to insert the balloon – 1.5cm as opposed to 1cm. A 1.5cm incision will be used for all participants, which is a very small change from standard care and is very unlikely to have a negative effect on any participant. Incisions will therefore be the same for both groups.

The operation note will be blinded to prevent accidental unblinding of the patient (for example, in the discharge information, or during post-operative physiotherapy). A standard recommended operation note template will be given to all sites adjusted to fit their local operation note systems. The details of the operation related to the balloon will be recorded in a secure online form easily accessible to the surgeon.

Unblinding may very rarely be required in an emergency situation, such as an overnight admission for suspected post-operative infection. Unblinding will be performed only by NHS staff in an emergency situation, by using a pre-defined web-based system, from a link inserted in the operation note. A two-way secure verification process will be performed using email, and an access code will be emailed only to an active NHS email address. A full explanation of the clinical circumstances and the need for access to data will be requested by the trial team for audit and monitoring purposes from the person who performed the unblinding, and the principal investigator (PI) for the site will be informed. The system has been designed and tested by the WCTU programming team to ensure that it is both secure and fully functional.

For peer review only - http://bmjopen.bmj.com/site/about/guidelines.xhtml

Participants will be asked at the 12 month time point, after collection of the primary outcome, if they were aware of their allocation.

End of trial

The trial will end when all participants have completed their 24 month follow-up, but the results will be published after the 12 month outcomes have been analysed, with a secondary report after collection of 24 month outcomes.

The trial may be stopped prematurely if: 1) mandated by the Ethics Committee; 2) There is an unexpected major safety concern; 3) Following recommendations from the Data Monitoring Committee (DMC) or Trial Steering Committee (TSC), which may include meeting the adaptive design boundaries, or 4) Funding for the trial ceases

Once a decision to stop has been taken, those within a short waiting time to surgery may be randomised at the discretion of the DMC and TSC, but after that participants will be treated according to the judgement of their clinician.

MRI Sub-study

Participants selected for inclusion in the MRI sub-study will undergo two research MRI scans at eight weeks and six months post intervention. MRI scans were preferred to x-rays for this purpose as measures taken from x-rays would be prone to error, primarily due to variation in the angle between the shoulder joint and the beam of the x-ray. This will assess the mechanism of action of the balloon, when the balloons are likely to be still inflated (but when acute post-operative pain has subsided), and when they are likely to have fully deflated, to see if the proposed mechanism for ongoing improvement is maintained. Passive imaging alone will not be adequate to demonstrate the function of the balloon and imaging will also need to be performed when the deltoid muscle is active, producing a proximally directed force on the humerus.(53, 54)

For this study we have developed (and piloted) a novel dynamic approach to assessing the function of the rotator cuff using MRI imaging under a mild deltoid load, to specifically assess the mechanism of the balloon. A separate paper on the pilot study for the trial is in preparation and will describe these methods in more detail.

All participants will be eligible (unless they have a contra-indication to MRI, or do not want to take part in the sub-study), but the sub-study will only be undertaken at a proportion of participating sites. The primary outcome will be the minimum acromio-humeral distance on the ‘deltoid-active’ coronal sequences at six months, a reliable and proven measure. (55-57) The balloon is expected to have deflated by 6 months.(58) Secondary measures will be acromio-humeral distance on passive images, and the change in acromio-humeral distance between active and passive images. The position of the balloon will be assessed to check for migration and consistency of placement relative to the acromion.

For peer review only - http://bmjopen.bmj.com/site/about/guidelines.xhtml

Adverse Events (AE), Adverse Device Events (ADE), Serious Adverse Device Effects (SADE)

Adverse events (AE), serious adverse events (SAEs), adverse device effects (ADE) and serious adverse device effects (SADE) will be defined using standard accepted criteria. An Unanticipated Serious Adverse Device Effect (USADE) is defined as a SADE which by its nature, incidence, severity or outcome has not been identified in the current version of the risk analysis report.

For the purposes of this trial AEs will be recorded for any participant where it is thought there may be a relationship between the trial interventions or the condition being studied (in this case, any shoulder condition, or related to the anaesthetic). These include device specific complications such as balloon migration, which will be recorded if it is identified by clinical teams from their normal practice.

All SAEs SADEs and USADEs occurring from the time of randomisation until 12 months post-randomisation must be communicated to the sponsor within 24 hours of the research staff becoming aware of the event. Events will be followed up until the event has resolved or a final outcome has been reached.

Where participants have been lost to follow up at or beyond the 12 month time point, and data on adverse events can therefore not be recorded, the participants General Practitioner (GP) will be contacted and a short form requesting any information or health record that could be an adverse event will be requested from the GP, as well as confirmation of the current contact details of the participant.

STATISTICAL ANALYSIS

Power and sample size

The initial sample size calculations were based on the Constant Score. The target difference that was chosen for the Constant Score was 10 units, as has been widely used for other trials, and the standard deviation was taken to be 20, giving a moderate standardised mean difference of 0.5.(17, 23, 59, 60) Anchor-based studies have estimated the target difference for the Oxford Shoulder Score as 6 and a standard deviation of 12 has been observed in multiple studies, therefore a moderate standardised mean difference of 0.5 remains appropriate.(41-43, 60)

For a costly invasive procedure of this nature a smaller standardised mean difference is unlikely to be considered worthwhile. For a power of 90% and a (two-sided) type I error rate of 5% a study with a conventional fixed design (i.e. with no possibility of stopping early), assuming an approximate normal distribution for the score data, would require 170 participants.

In order to assess the design characteristics and estimate the required sample size for the planned adaptive design, we undertook a large simulation study (see Parsons et al.(38)). As we expect correlations between time points (based on Karthikeyan et al 2010) and effect sizes to be equivalent between the Constant Score and Oxford Scores, these simulations remain valid, despite the change of primary outcome score.(41, 60) The simulations showed

For peer review only - http://bmjopen.bmj.com/site/about/guidelines.xhtml

that an adaptive design that allowed the possibility of early stopping for efficacy and/or futility, was feasible for the START:REACTS study.

Based on an assumed modest correlation between three and six month Oxford Shoulder Scores and 12 month Oxford Shoulder Scores equal to 0.5, and standard deviations of 12 for both three and six month scores, the simulations for the selected adaptive design, indicated that 188 participants would be sufficient to detect a 6 point difference in Oxford Shoulder Score between treatment arms with 90% power, and 5% (two-sided) type I error rate. Allowing for 15% loss to follow-up, whilst striving to keep this below 10%, gives a maximum study sample size of 221.

The sample size for MRI sub-study is based on the study by Gumina *et al.*(55)*,* where the minimum acromio-humeral distance had a standard deviation of 1.72mm. To observe a minimum important difference of 1.5mm (larger than the minimum detectable change of 1.3mm established elsewhere(61)) with an alpha of 0.05 at 80% power, assuming a loss to follow-up at 6 months of 20%, requires 56 participants for the MRI sub-study.

Statistical analysis plan

All data will be analysed and reported in accordance with the Consolidated Standards of Reporting Trials (CONSORT) guidelines.(62, 63). A detailed statistical analysis plan and a data sharing plan will be agreed with the DMC prior to any formal analyses being conducted.

Baseline data will be summarised to check comparability between treatment arms. Standard statistical summaries (e.g. means and standard deviations, dependent on data type) will be presented for the primary outcome measure and all secondary outcome measures.

The primary analysis will investigate differences in the Oxford Shoulder Score 12 months after surgery between the two treatment groups. It will be conducted on an intention-to-treat basis following the methods, test statistics and boundaries described by Parsons *et al.*(38) To preserve the integrity of the study, the exact boundaries used for testing are given in the adaptive charter known only to the study team and independent DMC. Briefly, if the study recruits to target, the method set out by Parsons *et al.*(38) will be used to calculate the boundaries and if the study stops early, testing will proceed using boundaries calculated by the deletion method for overrunning analysis, described by Whitehead, with clinical inferences following directly from widely used methods for unbiased estimates and confidence intervals in group sequential trials. (64-66)

The primary analysis will be augmented by calculating adjusted estimates of treatment group differences (with 95% confidence intervals) for the Oxford Shoulder Score using a mixed-effects model. The mixed-effects model will include a random effect for the recruiting centre, and fixed effects for the variables of interest included in the minimisation algorithm, patient age, gender and size of tear. In addition, subject to the observed data, sensitivity analyses such as per-protocol analyses or multiple imputation will also be undertaken. If undertaken, all multiple imputation assumptions will be justified and reported. Estimates of efficacy for the other outcome measures will follow this approach to analysis.

For peer review only - http://bmjopen.bmj.com/site/about/guidelines.xhtml

Pre-specified sub-group analyses will be undertaken to assess whether there is evidence that the intervention effect differs with respect to:

- The size of the rotator cuff tear as measured at the start of surgery, defined as large or massive cuff tear (≥3cm) or moderate to small (<3cm).
- Gender
- Age (>70 or <70)

These have been chosen as they are either important to the function of the intervention (cuff tear size) or to interpretation (gender and age). The subgroup analyses will follow the methods described for the mixed effects model of the Oxford Shoulder Score above, with additional interaction terms incorporated into the mixed-effects regression model to assess the level of support for these hypotheses. The study is not powered to formally test these hypotheses, so they will be reported as exploratory analyses only, and as subsidiary to the analysis reporting the main effects of the intervention in the full study population(67).

Interim analyses will only be undertaken following the principles laid out by Parsons et al.(38) Details of the timings or settings for the interim analyses will be treated as confidential information, so as not to prejudice the outcome of the trial based on the decision to stop the study or proceed, but will be recorded in the DMC minutes and on date-stamped internal documents. If a decision to stop the study early is made (for reasons other than safety), it will not be communicated outside of the DMC closed meeting whether this is for efficacy or futility.

HEALTH ECONOMIC EVALUATION

The economic component will include a standard health policy-relevant economic analysis and an exploration of how early economic data can be used to support decision making through the use of an adaptive design.

A prospective economic evaluation will be integrated into the trial and adhere to the recommendations of the NICE Reference Case.(68) Mechanisms of missingness of data will be explored and multiple imputation methods will be applied to impute missing data. Imputation sets will be used in bivariate analysis of costs and QALYs to generate incremental cost per QALY estimates and confidence regions.(69-72) It is anticipated that incremental costs and benefits will be captured within the trial and that extrapolated modelling will not be required.

Relatively little research has been conducted on how economic data available at interim time points can inform interim decision making. Currently, methods used are based mainly on net monetary benefit (NMB) approaches using value of information (VOI) methods (73, 74). We will compare various methods using economic data (costs and QALYs) and clinical data to evaluate the practical implications and operating characteristics of stopping a trial early based on cost-effectiveness data alone, efficacy data alone or a combination of cost-effectiveness and efficacy. We will use the trial to evaluate putative analytic methods, as set out within a prospectively written health economic analysis plan and carry out parallel interim analyses, separate from the real trial analyses, exploring how interim decisions might have been influenced. As such health economic decision rules for adaptive designs

For peer review only - http://bmjopen.bmj.com/site/about/guidelines.xhtml

are less widely understood and used in the literature, the properties of these methods will be developed further for future trials.

DISSEMINATION AND PUBLICATION

The trial will be reported in accordance with the Consolidated Standards of Reporting Trials (CONSORT) guidelines and the 2018 CONSORT extension for adaptive trials.(62, 63)

The results will be submitted to a high impact peer-reviewed journal with authorship following ICMJE recommendations, and will be presented at national and international meetings such as the British Elbow and Shoulder Society, the British Orthopaedic Association, and the American Academy of Orthopaedic Surgeons. The investigators will share data (with associated coding library) used in developing the results on request to the chief investigator, subject to a formal data sharing agreement being in place. We will only share anonymised record level data with those who have received ethical clearance from their host institution.

To inform patients and the public, we intend to produce a lay summary, which will be made available in the trial hospitals and to patients involved in the trial. In addition, we will publicise the work through social media outlets (e.g. Facebook and twitter) as well as websites such as Patient.co.uk.

For peer review only

For peer review only - http://bmjopen.bmj.com/site/about/guidelines.xhtml

Author Contributions. All the authors helped to developed the trial protocol and all have contributed to the writing of the manuscript and approved its final version. TL and SD first identified the clinical question. The clinical question and adaptive deign components were developed by AM, TL, SD, MU, HP, NP, NS, RK, JM, IK, and SG in close collaboration with JF throughout. These same co-authors developed the study methodology with EGM, JB, KM, HB, and CH. CH leads the MRI sub-study with AM. This protocol was written following the SPIRIT protocol guidance. Andrew Metcalfe is the chief investigator and main grant holder for this study.

Funding. This project (project reference 16.61.18) is funded by the Efficacy and Mechanism Evaluation (EME) Programme, an MRC and NIHR partnership. The views expressed in this publication are those of the author(s) and not necessarily those of the MRC, NIHR or the Department of Health and Social Care.

Sponsorship. The trial is co-sponsored by the University of Warwick and University Hospitals Coventry and Warwickshire NHS trust.

Competing interests. Stryker are providing 50 free InSpace balloons for the study, to offset some of the costs of delivery of the study in participating hospitals. Contracts are in place to ensure the full independence of the trial team with regard to study design and delivery, analysis and reporting of results, aligning to the NIHR standard agreement. The chief investigator and multiple co-authors (HP, EGM, JB, JF, JM, CH. MU) are applicants on another NIHR grant with similar contractual relationships with Stryker to support treatment costs but not research costs, in which the full independence of the trial team is protected by mutually agreed contracts.

MU was Chair of the NICE accreditation advisory committee until March 2017 for which he received a fee. He is chief investigator or co-investigator on multiple previous and current research grants from the National Institute for Health Research. He is an NIHR Senior Investigator. He is an editor of the NIHR journal series, and a member of the NIHR Journal Editors Group, for which he receives a fee.

There are no other conflicts of interest to declare

Provenance and peer review. Not commissioned; peer reviewed for ethical and funding approval prior to submission.

Open Access. This is an Open Access article distributed in accordance with the terms of the Creative Commons Attribution (CC BY 4.0) license, which permits others to distribute, remix, adapt and build upon this work, for commercial use, provided the original work is properly cited. See: http://creativecommons.org/licenses/by/4.0/

For peer review only - http://bmjopen.bmj.com/site/about/guidelines.xhtml

For peer review only

REFERENCES

1. Carnes D, Parsons S, Ashby D, Breen A, Foster NE, Pincus T, et al. Chronic musculoskeletal pain rarely presents in a single body site: Results from a UK population study. Rheumatology. 2007;46:1168-70.
2. Ostor AJ, Richards CA, Prevost AT, Speed CA, Hazleman BL. Diagnosis and relation to general health of shoulder disorders presenting to primary care. Rheumatology (Oxford). 2005;44(6):800-5.
3. Teunis T, Lubberts B, Reilly BT, Ring D. A systematic review and pooled analysis of the prevalence of rotator cuff disease with increasing age. J Shoulder Elbow Surg. 2014;23(12):1913-21.
4. Urwin M, Symmons D, Allison T, Brammah T, Busby H, Roxby M, et al. Estimating the burden of musculoskeletal disorders in the community: the comparative prevalence of symptoms at different anatomical sites, and the relation to social deprivation. Ann Rheum Dis. 1998;57(11):649-55.
5. Largacha M, Parsons IMt, Campbell B, Titelman RM, Smith KL, Matsen F, 3rd. Deficits in shoulder function and general health associated with sixteen common shoulder diagnoses: a study of 2674 patients. J Shoulder Elbow Surg. 2006;15(1):30-9.
6. Minns Lowe CJ, Moser J, Barker K. Living with a symptomatic rotator cuff tear 'bad days, bad nights': a qualitative study. BMC Musculoskelet Disord. 2014;15:228.
7. Virta L, Joranger P, Brox JI, Eriksson R. Costs of shoulder pain and resource use in primary health care: a cost-of-illness study in Sweden. BMC Musculoskelet Disord. 2012;13:17.
8. Whittle S, Buchbinder R. In the clinic. Rotator cuff disease. Ann Intern Med. 2015;162(1):ITC1-15.
9. Carr AJ, Cooper CD, Campbell MK, Rees JL, Moser J, Beard DJ, et al. Clinical effectiveness and cost-effectiveness of open and arthroscopic rotator cuff repair [the UK Rotator Cuff Surgery (UKUFF) randomised trial]. Health Technol Assess. 2015;19(80):1-218.
10. Acevedo DC, Paxton ES, Williams GR, Abboud JA. A Survey of Expert Opinion Regarding Rotator Cuff Repair. J Bone Joint Surg Am. 2014;96(14):e123.
11. Anley CM, Chan SK, Snow M. Arthroscopic treatment options for irreparable rotator cuff tears of the shoulder. World J Orthop. 2014;5(5):557-65.
12. Leung B, Horodyski M, Struk AM, Wright TW. Functional outcome of hemiarthroplasty compared with reverse total shoulder arthroplasty in the treatment of rotator cuff tear arthropathy. J Shoulder Elbow Surg. 2012;21(3):319-23.
13. Harreld KL, Puskas BL, Frankle M. Massive rotator cuff tears without arthropathy: when to consider reverse shoulder arthroplasty. J Bone Joint Surg Am. 2011;93(10):973-84.
14. Mulieri P, Dunning P, Klein S, Pupello D, Frankle M. Reverse shoulder arthroplasty for the treatment of irreparable rotator cuff tear without glenohumeral arthritis. J Bone Joint Surg Am. 2010;92(15):2544-56.
15. Rockwood CA, Jr., Williams GR, Jr., Burkhead WZ, Jr. Debridement of degenerative, irreparable lesions of the rotator cuff. J Bone Joint Surg Am. 1995;77(6):857-66.
16. Liem D, Lengers N, Dedy N, Poetzl W, Steinbeck J, Marquardt B. Arthroscopic debridement of massive irreparable rotator cuff tears. Arthroscopy. 2008;24(7):743-8.
17. Kukkonen J, Joukainen A, Lehtinen J, Mattila KT, Tuominen EK, Kauko T, et al. Treatment of non-traumatic rotator cuff tears: A randomised controlled trial with one-year clinical results. Bone Joint J. 2014;96-B(1):75-81.

For peer review only - http://bmjopen.bmj.com/site/about/guidelines.xhtml

18. National Institute for Health and Care Excellence. Biodegradable subacromial spacer insertion for rotator cuff tears. Interv Proced Guid IPG558 [Internet]. 2016 11/04/17. Available from: https://www.nice.org.uk/guidance/ipg558.
19. Savarese E, Romeo R. New solution for massive, irreparable rotator cuff tears: the subacromial "biodegradable spacer". Arthrosc Tech. 2012;1(1):e69-74.
20. Szollosy G, Rosso C, Fogerty S, Petkin K, Lafosse L. Subacromial spacer placement for protection of rotator cuff repair. Arthrosc Tech. 2014;3(5):e605-9.
21. Ramot Y, Nyska A, Markovitz E, Dekel A, Klaiman G, Zada MH, et al. Long-term Local and Systemic Safety of Poly(L-lactide-co-epsilon-caprolactone) after Subcutaneous and Intra-articular Implantation in Rats. Toxicol Pathol. 2015;43(8):1127-40.
22. Senekovic V, Poberaj B, Kovacic L, Mikek M, Adar E, Dekel A. Prospective clinical study of a novel biodegradable sub-acromial spacer in treatment of massive irreparable rotator cuff tears. Eur J Orthop Surg Traumatol. 2013;23(3):311-6.
23. Senekovic V, Poberaj B, Kovacic L, Mikek M, Adar E, Markovitz E, et al. The biodegradable spacer as a novel treatment modality for massive rotator cuff tears: a prospective study with 5-year follow-up. Archives of Orthopaedic and Trauma Surgery. 2016;137:95-103.
24. Bakti N, Wlliamson M, Gulihar M, Singh R. Patient reported functional outcome following subacromial spacer insertion for irrepairable rotator cuff tears: a 12 month review. BJJ Orthop Proc. In press.
25. Henderson D, Simeson K, Venkateswaran B. Sub-acromial spacer vs. partial rotator cuff repair; A single surgeon cohort study. BJJ Orthop Proc. In press.
26. Prasad VR, Fung M, Borowsky KA, Tolat AR, Singh B. Outcomes of inspace balloon arthroplasty for irreparable cuff tear at two years: a longitudinal study. Bone & Joint Journal Orthopaedic Proceedings Supplement. 2016;98-B(SUPP 13):1-.
27. Holschen M, Brand F, Agneskirchner JD. Subacromial spacer implantation for massive rotator cuff tears. Obere Extremität. 2017;12:38-45.
28. Costa ML, Achten J, Parsons NR, Edlin RP, Foguet P, Prakash U, et al. Total hip arthroplasty versus resurfacing arthroplasty in the treatment of patients with arthritis of the hip joint: single centre, parallel group, assessor blinded, randomised controlled trial. BMJ. 2012;344:e2147.
29. Costa ML, Achten J, Parsons NR, Rangan A, Griffin D, Tubeuf S, et al. Percutaneous fixation with Kirschner wires versus volar locking plate fixation in adults with dorsally displaced fracture of distal radius: randomised controlled trial. BMJ. 2014;349:g4807.
30. McCulloch P. Developing appropriate methodology for the study of surgical techniques. Journal of the Royal Society of Medicine. 2009;102:51-5.
31. Achten J, Parsons NR, Bruce J, Petrou S, Tutton E, Willett K, et al. Protocol for a randomised controlled trial of standard wound management versus negative pressure wound therapy in the treatment of adult patients with an open fracture of the lower limb: UK Wound management of Open Lower Limb Fractures (UK WOLFF). BMJ Open. 2015;5(9):e009087.
32. Bhatt DL, Mehta C. Adaptive Designs for Clinical Trials. N Engl J Med. 2016;375(1):65-74.
33. U.S. Department of Health and Human Services Food and Drug Administration. Adaptive Designs for Medical Device Clinical Studies final guidance2016 21/04/2017. Available from:

For peer review only - http://bmjopen.bmj.com/site/about/guidelines.xhtml

https://www.fda.gov/downloads/medicaldevices/deviceregulationandguidance/guidancedocuments/ucm446729.pdf.
34. Dimairo M, Boote J, Julious SA, Nicholl JP, Todd S. Missing steps in a staircase: a qualitative study of the perspectives of key stakeholders on the use of adaptive designs in confirmatory trials. Trials. 2015;16:430.
35. Campbell G, Yue LQ. Statistical innovations in the medical device world sparked by the FDA. J Biopharm Stat. 2016;26(1):3-16.
36. Elsasser A, Regnstrom J, Vetter T, Koenig F, Hemmings RJ, Greco M, et al. Adaptive clinical trial designs for European marketing authorization: a survey of scientific advice letters from the European Medicines Agency. Trials. 2014;15:383.
37. Kairalla Ja, Coffey CS, Thomann Ma, Muller KE. Adaptive trial designs: a review of barriers and opportunities. Trials. 2012;13:145.
38. Parsons N, Stallard N, Parsons H, Wells P, Underwood M, Mason J, et al. An adaptive two-arm clinical trial using early endpoints to inform decision making: design for a study of sub-acromial spacers for repair of rotator cuff tendon tear Trials. 2019;20:694: https://doi.org/10.1186/s13063-019-3708-6
39. Dawson J, Fitzpatrick R, Carr A. Questionnaire on the perceptions of patients about shoulder surgery. The Journal of bone and joint surgery British volume. 1996;78:593-600.
40. Dawson J, Rogers K, Fitzpatrick R, Carr A. The Oxford shoulder score revisited. Arch Orthop Trauma Surg. 2009;129(1):119-23.
41. Christiansen DHH, Frost P, Falla D, Haahr JP, Frich LH, Svendsen SW. Responsiveness and Minimal Clinically Important Change: A Comparison Between 2 Shoulder Outcome Measures. Journal of Orthopaedic and Sports Physical Therapy. 2015;45:620-5.
42. Beard DJ, Rees JL, Cook JA, Rombach I, Cooper C, Merritt N, et al. Arthroscopic subacromial decompression for subacromial shoulder pain (CSAW): a multicentre, pragmatic, parallel group, placebo-controlled, three-group, randomised surgical trial. Lancet. 2018;391(10118):329-38.
43. van Kampen DA, Willems WJ, van Beers LW, Castelein RM, Scholtes VA, Terwee CB. Determination and comparison of the smallest detectable change (SDC) and the minimal important change (MIC) of four-shoulder patient-reported outcome measures (PROMs). J Orthop Surg Res. 2013;8:40.
44. Khatri C, Ahmed I, Parsons H, Smith NA, Lawrence TM, Modi CS, et al. The Natural History of Full-Thickness Rotator Cuff Tears in Randomized Controlled Trials: A Systematic Review and Meta-analysis. The American journal of sports medicine. 2019;47(7):1734-43.
45. Constant CR, Gerber C, Emery RJ, Sojbjerg JO, Gohlke F, Boileau P. A review of the Constant score: modifications and guidelines for its use. J Shoulder Elbow Surg. 2008;17(2):355-61.
46. Constant CR, Murley AH. A clinical method of functional assessment of the shoulder. Clin Orthop Relat Res. 1987(214):160-4.
47. Moeller AD, Thorsen RR, Torabi TP, Bjoerkman AS, Christensen EH, Maribo T, et al. The Danish version of the modified Constant-Murley shoulder score: reliability, agreement, and construct validity. J Orthop Sports Phys Ther. 2014;44(5):336-40.
48. Holtby R, Razmjou H. Measurement properties of the Western Ontario rotator cuff outcome measure: a preliminary report. J Shoulder Elbow Surg. 2005;14(5):506-10.
49. Brooks R. EuroQol: the current state of play. Health Policy. 1996;37(1):53-72.

For peer review only - http://bmjopen.bmj.com/site/about/guidelines.xhtml

50. Janssen MF, Pickard AS, Golicki D, Gudex C, Niewada M, Scalone L, et al. Measurement properties of the EQ-5D-5L compared to the EQ-5D-3L across eight patient groups: a multi-country study. Qual Life Res. 2013;22(7):1717-27.
51. Kamper SJ, Maher CG, Mackay G. Global rating of change scales: a review of strengths and weaknesses and considerations for design. J Man Manip Ther. 2009;17(3):163-70.
52. Kellgren JH, Lawrence JS. Radiological assessment of osteo-arthrosis. Ann Rheum Dis. 1957;16(4):494-502.
53. Burkhart SS. Reconciling the paradox of rotator cuff repair versus debridement: a unified biomechanical rationale for the treatment of rotator cuff tears. Arthroscopy. 1994;10(1):4-19.
54. Huegel J, Williams AA, Soslowsky LJ. Rotator cuff biology and biomechanics: a review of normal and pathological conditions. Current rheumatology reports. 2015;17:476.
55. Gumina S, Arceri V, Fagnani C, Venditto T, Catalano C, Candela V, et al. Subacromial Space Width: Does Overuse or Genetics Play a Greater Role in Determining It? An MRI Study on Elderly Twins. J Bone Joint Surg Am. 2015;97(20):1647-52.
56. McCreesh KM, Crotty JM, Lewis JS. Acromiohumeral distance measurement in rotator cuff tendinopathy: is there a reliable, clinically applicable method? A systematic review. Br J Sports Med. 2015;49(5):298-305.
57. Tempelaere C, Pierrart J, Lefevre-Colau MM, Vuillemin V, Cuenod CA, Hansen U, et al. Dynamic Three-Dimensional Shoulder Mri during Active Motion for Investigation of Rotator Cuff Diseases. PLoS One. 2016;11(7):e0158563.
58. Ricci M, Vecchini E, Bonfante E, Micheloni GM, Berti M, Schenal G, et al. A clinical and radiological study of biodegradable subacromial spacer in the treatment of massive irreparable rotator cuff tears. Acta Biomed. 2017;88(4S):75-80.
59. Haahr JP, Ostergaard S, Dalsgaard J, Norup K, Frost P, Lausen S, et al. Exercises versus arthroscopic decompression in patients with subacromial impingement: a randomised, controlled study in 90 cases with a one year follow up. Ann Rheum Dis. 2005;64(5):760-4.
60. Karthikeyan S, Kwong HT, Upadhyay PK, Parsons N, Drew SJ, Griffin D. A double-blind randomised controlled study comparing subacromial injection of tenoxicam or methylprednisolone in patients with subacromial impingement. J Bone Joint Surg Br. 2010;92(1):77-82.
61. Kalra N, Seitz AL, Boardman ND, 3rd, Michener LA. Effect of posture on acromiohumeral distance with arm elevation in subjects with and without rotator cuff disease using ultrasonography. J Orthop Sports Phys Ther. 2010;40(10):633-40.
62. Moher D, Schulz KF, Altman DG. The CONSORT statement: revised recommendations for improving the quality of reports of parallel-group randomised trials. The Lancet. 2001;357:1191-4.
63. Dimairo M, Coates E, Pallmann P, Todd S, Julious SA, Jaki T, et al. Development process of a consensus-driven CONSORT extension for randomised trials using an adaptive design. BMC Med. 2018;16(1):210.
64. Whitehead J. Overrunning and underrunning in sequential clinical trials. Control Clin Trials. 1992;13(2):106-21.
65. Liu A, Hall WJ. Unbiased estimation following a group sequential test. . Biometrika. 1999;86:71-8.
66. Todd S, Whitehead J, Facey KM. Point and interval estimation following a sequential clinical trial. . Biometrika. 1996; 83:453-61.

For peer review only - http://bmjopen.bmj.com/site/about/guidelines.xhtml

67. Pincus T, Miles C, Froud R, Underwood M, Carnes D, Taylor SJ. Methodological criteria for the assessment of moderators in systematic reviews of randomised controlled trials: a consensus study. BMC Med Res Methodol. 2011;11:14.
68. National Institute for Health and Care Excellence. Guide to the methods of technology appraisal 2013. Process and methods [PMG9] [Internet]. 2013 11/04/17. Available from: https://www.nice.org.uk/process/pmg9/resources/guide-to-the-methods-of-technology-appraisal-2013-pdf-2007975843781.
69. Manca A, Hawkins N, Sculpher MJ. Estimating mean QALYs in trial-based cost-effectiveness analysis: the importance of controlling for baseline utility. Health Econ. 2005;14(5):487-96.
70. Sterne JA, White IR, Carlin JB, Spratt M, Royston P, Kenward MG, et al. Multiple imputation for missing data in epidemiological and clinical research: potential and pitfalls. BMJ. 2009;338:b2393.
71. White IR, Horton NJ, Carpenter J, Pocock SJ. Strategy for intention to treat analysis in randomised trials with missing outcome data. BMJ. 2011;342:d40.
72. White IR, Royston P, Wood AM. Multiple imputation using chained equations: Issues and guidance for practice. Stat Med. 2011;30(4):377-99.
73. Chen MH, Willan AR. Determining optimal sample sizes for multistage adaptive randomized clinical trials from an industry perspective using value of information methods. Clin Trials. 2013;10(1):54-62.
74. Willan A, Kowgier M. Determining optimal sample sizes for multi-stage randomized clinical trials using value of information methods. Clin Trials. 2008;5(4):289-300.

For peer review only

For peer review only - http://bmjopen.bmj.com/site/about/guidelines.xhtml

Reporting checklist for protocol of a clinical trial.

Based on the SPIRIT guidelines.

Instructions to authors

Complete this checklist by entering the page numbers from your manuscript where readers will find each of the items listed below.

Your article may not currently address all the items on the checklist. Please modify your text to include the missing information. If you are certain that an item does not apply, please write "n/a" and provide a short explanation.

Upload your completed checklist as an extra file when you submit to a journal.

In your methods section, say that you used the SPIRITreporting guidelines, and cite them as:

Chan A-W, Tetzlaff JM, Altman DG, Laupacis A, Gøtzsche PC, Krleža-Jerić K, Hróbjartsson A, Mann H, Dickersin K, Berlin J, Doré C, Parulekar W, Summerskill W, Groves T, Schulz K, Sox H, Rockhold FW, Rennie D, Moher D. SPIRIT 2013 Statement: Defining standard protocol items for clinical trials. Ann Intern Med. 2013;158(3):200-207

		Reporting Item	Page Number
**Administrative information**			
Title	#1	Descriptive title identifying the study design, population, interventions, and, if applicable, trial acronym	1
Trial registration	#2a	Trial identifier and registry name. If not yet registered, name of intended registry	2
Trial registration: data set	#2b	All items from the World Health Organization Trial Registration Data Set	2
Protocol version	#3	Date and version identifier	n/a
Funding	#4	Sources and types of financial, material, and other support	16
Roles and responsibilities: contributorship	#5a	Names, affiliations, and roles of protocol contributors	1,16

For peer review only

Roles and responsibilities: sponsor contact information	#5b	Name and contact information for the trial sponsor	16
Roles and responsibilities: sponsor and funder	#5c	Role of study sponsor and funders, if any, in study design; collection, management, analysis, and interpretation of data; writing of the report; and the decision to submit the report for publication, including whether they will have ultimate authority over any of these activities	16
Roles and responsibilities: committees	#5d	Composition, roles, and responsibilities of the coordinating centre, steering committee, endpoint adjudication committee, data management team, and other individuals or groups overseeing the trial, if applicable (see Item 21a for data monitoring committee)	6,7
**Introduction**			
Background and rationale	#6a	Description of research question and justification for undertaking the trial, including summary of relevant studies (published and unpublished) examining benefits and harms for each intervention	4,5
Background and rationale: choice of comparators	#6b	Explanation for choice of comparators	4,5
Objectives	#7	Specific objectives or hypotheses	6
Trial design	#8	Description of trial design including type of trial (eg, parallel group, crossover, factorial, single group), allocation ratio, and framework (eg, superiority, equivalence, non-inferiority, exploratory)	6
**Methods: Participants, interventions, and outcomes**			
Study setting	#9	Description of study settings (eg, community clinic, academic hospital) and list of countries where data will be	6,9

For peer review only

		collected. Reference to where list of study sites can be obtained	
Eligibility criteria	#10	Inclusion and exclusion criteria for participants. If applicable, eligibility criteria for study centres and individuals who will perform the interventions (eg, surgeons, psychotherapists)	8,9
Interventions: description	#11a	Interventions for each group with sufficient detail to allow replication, including how and when they will be administered	10
Interventions: modifications	#11b	Criteria for discontinuing or modifying allocated interventions for a given trial participant (eg, drug dose change in response to harms, participant request, or improving / worsening disease)	n/a
Interventions: adherance	#11c	Strategies to improve adherence to intervention protocols, and any procedures for monitoring adherence (eg, drug tablet return; laboratory tests)	10
Interventions: concomitant care	#11d	Relevant concomitant care and interventions that are permitted or prohibited during the trial	10
Outcomes	#12	Primary, secondary, and other outcomes, including the specific measurement variable (eg, systolic blood pressure), analysis metric (eg, change from baseline, final value, time to event), method of aggregation (eg, median, proportion), and time point for each outcome. Explanation of the clinical relevance of chosen efficacy and harm outcomes is strongly recommended	7
Participant timeline	#13	Time schedule of enrolment, interventions (including any run-ins and washouts), assessments, and visits for participants. A schematic diagram is highly recommended (see Figure)	6,7,9,11
Sample size	#14	Estimated number of participants needed to achieve study objectives and how it was determined, including clinical and statistical assumptions supporting any sample size calculations	12,13
Recruitment	#15	Strategies for achieving adequate participant enrolment to reach target sample size	9

For peer review only

**Methods: Assignment of interventions (for controlled trials)**			
Allocation: sequence generation	#16a	Method of generating the allocation sequence (eg, computer-generated random numbers), and list of any factors for stratification. To reduce predictability of a random sequence, details of any planned restriction (eg, blocking) should be provided in a separate document that is unavailable to those who enrol participants or assign interventions	9
Allocation concealment mechanism	#16b	Mechanism of implementing the allocation sequence (eg, central telephone; sequentially numbered, opaque, sealed envelopes), describing any steps to conceal the sequence until interventions are assigned	9
Allocation: implementation	#16c	Who will generate the allocation sequence, who will enrol participants, and who will assign participants to interventions	9,10,11
Blinding (masking)	#17a	Who will be blinded after assignment to interventions (eg, trial participants, care providers, outcome assessors, data analysts), and how	10,11
Blinding (masking): emergency unblinding	#17b	If blinded, circumstances under which unblinding is permissible, and procedure for revealing a participant's allocated intervention during the trial	11
**Methods: Data collection, management, and analysis**			
Data collection plan	#18a	Plans for assessment and collection of outcome, baseline, and other trial data, including any related processes to promote data quality (eg, duplicate measurements, training of assessors) and a description of study instruments (eg, questionnaires, laboratory tests) along with their reliability and validity, if known. Reference to where data collection forms can be found, if not in the protocol	7,8,9,10,11,12

For peer review only

Data collection plan: retention	#18b	Plans to promote participant retention and complete follow-up, including list of any outcome data to be collected for participants who discontinue or deviate from intervention protocols	7,12
Data management	#19	Plans for data entry, coding, security, and storage, including any related processes to promote data quality (eg, double data entry; range checks for data values). Reference to where details of data management procedures can be found, if not in the protocol	8
Statistics: outcomes	#20a	Statistical methods for analysing primary and secondary outcomes. Reference to where other details of the statistical analysis plan can be found, if not in the protocol	13,14,15
Statistics: additional analyses	#20b	Methods for any additional analyses (eg, subgroup and adjusted analyses)	13,14,15
Statistics: analysis population and missing data	#20c	Definition of analysis population relating to protocol non-adherence (eg, as randomised analysis), and any statistical methods to handle missing data (eg, multiple imputation)	13,14,15
**Methods: Monitoring**			
Data monitoring: formal committee	#21a	Composition of data monitoring committee (DMC); summary of its role and reporting structure; statement of whether it is independent from the sponsor and competing interests; and reference to where further details about its charter can be found, if not in the protocol. Alternatively, an explanation of why a DMC is not needed	6,7,11,13,14
Data monitoring: interim analysis	#21b	Description of any interim analyses and stopping guidelines, including who will have access to these interim results and make the final decision to terminate the trial	11,14
Harms	#22	Plans for collecting, assessing, reporting, and managing solicited and spontaneously reported adverse events and other unintended effects of trial interventions or trial conduct	12
Auditing	#23	Frequency and procedures for auditing trial conduct, if any, and whether the process will be independent from investigators and the sponsor	7

For peer review only

**Ethics and dissemination**			
Research ethics approval	#24	Plans for seeking research ethics committee / institutional review board (REC / IRB) approval	7
Protocol amendments	#25	Plans for communicating important protocol modifications (eg, changes to eligibility criteria, outcomes, analyses) to relevant parties (eg, investigators, REC / IRBs, trial participants, trial registries, journals, regulators)	7
Consent or assent	#26a	Who will obtain informed consent or assent from potential trial participants or authorised surrogates, and how (see Item 32)	9
Consent or assent: ancillary studies	#26b	Additional consent provisions for collection and use of participant data and biological specimens in ancillary studies, if applicable	n/a
Confidentiality	#27	How personal information about potential and enrolled participants will be collected, shared, and maintained in order to protect confidentiality before, during, and after the trial	7,8,9,11,12
Declaration of interests	#28	Financial and other competing interests for principal investigators for the overall trial and each study site	16
Data access	#29	Statement of who will have access to the final trial dataset, and disclosure of contractual agreements that limit such access for investigators	13
Ancillary and post trial care	#30	Provisions, if any, for ancillary and post-trial care, and for compensation to those who suffer harm from trial participation	n/a
Dissemination policy: trial results	#31a	Plans for investigators and sponsor to communicate trial results to participants, healthcare professionals, the public, and other relevant groups (eg, via publication, reporting in results databases, or other data sharing arrangements), including any publication restrictions	15
Dissemination policy: authorship	#31b	Authorship eligibility guidelines and any intended use of professional writers	15

For peer review only

Dissemination policy: reproducible research	#31c	Plans, if any, for granting public access to the full protocol, participant-level dataset, and statistical code	n/a

Appendices

Informed consent materials	#32	Model consent form and other related documentation given to participants and authorised surrogates	n/a
Biological specimens	#33	Plans for collection, laboratory evaluation, and storage of biological specimens for genetic or molecular analysis in the current trial and for future use in ancillary studies, if applicable	n/a

Notes:

- 18a: 7,8,9,10,11,12
- 21a: 6,7,11,13,14
- 27: 7,8,9,11,12 The SPIRIT checklist is distributed under the terms of the Creative Commons Attribution License CC-BY-ND 3.0. This checklist was completed on 03. January 2020 using https://www.goodreports.org/, a tool made by the EQUATOR Network in collaboration with Penelope.ai

For peer review only
